# Virtual Screening, pharmacophore development and structure based similarity search to identify inhibitors against IdeR, a transcription factor of *Mycobacterium tuberculosis*

**DOI:** 10.1038/s41598-017-04748-9

**Published:** 2017-07-05

**Authors:** Akshay Rohilla, Garima Khare, Anil K. Tyagi

**Affiliations:** 1 Department of Biochemistry, University of Delhi South Campus, Benito Juarez road, New Delhi, 110021 India; 2 Vice Chancellor, Guru Gobind Singh Indraprastha University, Sector 16-C, Dwarka, New Delhi India

## Abstract

*ideR*, an essential gene of *Mycobacterium tuberculosis*, is an attractive drug target as its conditional knockout displayed attenuated growth phenotype *in vitro* and *in vivo*. To the best of our knowledge, no inhibitors of IdeR are identified. We carried out virtual screening of NCI database against the IdeR DNA binding domain followed by inhibition studies using EMSA. Nine compounds exhibited potent inhibition with NSC 281033 (I-20) and NSC 12453 (I-42) exhibiting IC_50_ values of 2 µg/ml and 1 µg/ml, respectively. We then attempted to optimize the leads firstly by structure based similarity search resulting in a class of inhibitors based on I-42 containing benzene sulfonic acid, 4-hydroxy-3-[(2-hydroxy-1-naphthalenyl) azo] scaffold with 4 molecules exhibiting IC_50_ ≤ 10 µg/ml. Secondly, optimization included development of energy based pharmacophore and screening of ZINC database followed by docking studies, yielding a molecule with IC_50_ of 60 µg/ml. More importantly, a five-point pharmacophore model provided insight into the features essential for IdeR inhibition. Five molecules with promising IC_50_ values also inhibited *M. tuberculosis* growth in broth culture with MIC_90_ ranging from 17.5 µg/ml to 100 µg/ml and negligible cytotoxicity in various cell lines. We believe our work opens up avenues for further optimization studies.

## Introduction

Despite the availability of an effective frontline chemotherapeutic regimen comprising of four frontline drugs namely isoniazid, pyrazinamide, rifampicin and ethambutol, the morbidity and mortality rates associated with TB patients remain very high. Moreover, the lengthy treatment of 6–9 months often leads to non-compliance by the patients leading to an inexorable increase in the multi drug resistant and extremely drug resistant strains of *Mycobacterium tuberculosis* ^1, 2^. Thus, in view of the current scenario, the urgent need to identify novel potent inhibitory molecules against *M. tuberculosis* cannot be overemphasized.

Iron is an essential element required for the activity of numerous proteins involved in redox reactions and electron transport chain^3^. Down regulation of iron storage proteins such as transferrin and ferritins remains one of the critical immune responses generated by the host against the invading pathogen^4–7^. In response to this, *M. tuberculosis* upregulates its iron acquisition machinery, which synthesizes small molecules known as mycobactins and carboxymycobactins that function as iron chelators^8, 9^. These molecules bind iron from the host proteins and with the help of various transporters, this iron is then transported to the *M. tuberculosis* cytosol, where it is utilized for many crucial processes^10–14^. Although, iron is an essential element, it is toxic, if present, in higher amounts. Excess iron can react with peroxides to form free radicals via fenton reaction leading to cellular toxicity^15^. Thus, the pathogen requires a tight regulation of the intracellular levels of iron, which in *M. tuberculosis* is performed by the transcription factor IdeR. In the conditions of iron sufficiency in *M. tuberculosis*, iron binds and activates intracellular IdeR, which then represses the iron acquisition machinery of the pathogen and activates the synthesis of iron storage proteins. In the case of iron deficiency, IdeR can no longer bind to the target promoter region resulting in the production of mycobactins and repression of the synthesis of iron storage proteins^16^.

A conditional knockout of *M. tuberculosis ideR* gene displayed an attenuated phenotype when grown *in vitro* and *in vivo* suggesting the importance of IdeR for the growth and survival of *M. tuberculosis* ^17^. Besides, IdeR also plays a crucial role in protecting the cells against the oxidative stress generated by the host. These properties make IdeR an attractive drug target^17^. A series of studies carried out by Hol *et al*. on the crystal structures of *M. tuberculosis* IdeR in monomer and DNA bound forms identified Ser 37, Pro 39 and Gln 43 as few of the residues crucial for the binding of IdeR to the DNA molecule^18–21^. IdeR consists of two metal binding sites 1 and 2 and a dimerization domain which helps in the dimerization of IdeR monomers when iron is bound at the high affinity metal binding site 1 with a K_d_ less than 0.5 µM Fe^2+^. IdeR also carries a DNA binding domain which undergoes a conformational change when iron binds at the metal binding site 2 with a relatively lower affinity for iron having a K_d_ of 9.5 µM^22^. The distance between Cα atom of Gln 43 (an important residue present at the DNA binding helix) to metal binding site 1 is 26.7 Å and metal binding site 2 is 19.5 Å as calculated by Pohl *et al*.^18^. As is evident from the relative distances, the two iron binding sites are far away from the DNA binding helix of the protein IdeR. Studies of the crystal structure determination, iron binding kinetics and molecular dynamics simulation^23^ of IdeR have provided insights into the binding mechanism of IdeR with iron and its cognate DNA sequences and have established a starting point for carrying out structure based identification of potent inhibitory molecules against IdeR.

In this study, we took a structure based approach which included virtual screening followed by Electrophoretic Mobility Shift Assay (EMSA) to evaluate the inhibitory potential of various shortlisted molecules against the DNA binding ability of IdeR. Subsequently, the leads obtained from this approach were further utilized for (a) structure based similarity search approach which resulted in 4-hydroxy-3-[(2-hydroxy-1-naphthalenyl) azo] scaffold bearing molecules with potent *in vitro* inhibition and (b) energy based pharmacophore model generation followed by docking study which yielded a molecule with IC_50_ of 60 µg/ml and also provided an insight into the critical features required for IdeR based inhibition. Further, the molecules were evaluated against the growth of *M. tuberculosis* in broth culture followed by cytotoxicity studies in macrophage, kidney and hepatic cell lines resulting in several molecules that can be employed as starting points for carrying out further structure activity relationship studies to inhibit IdeR.

## Results and Discussion

### *In silico* screening against the DNA binding domain of IdeR

**Figure 1:**
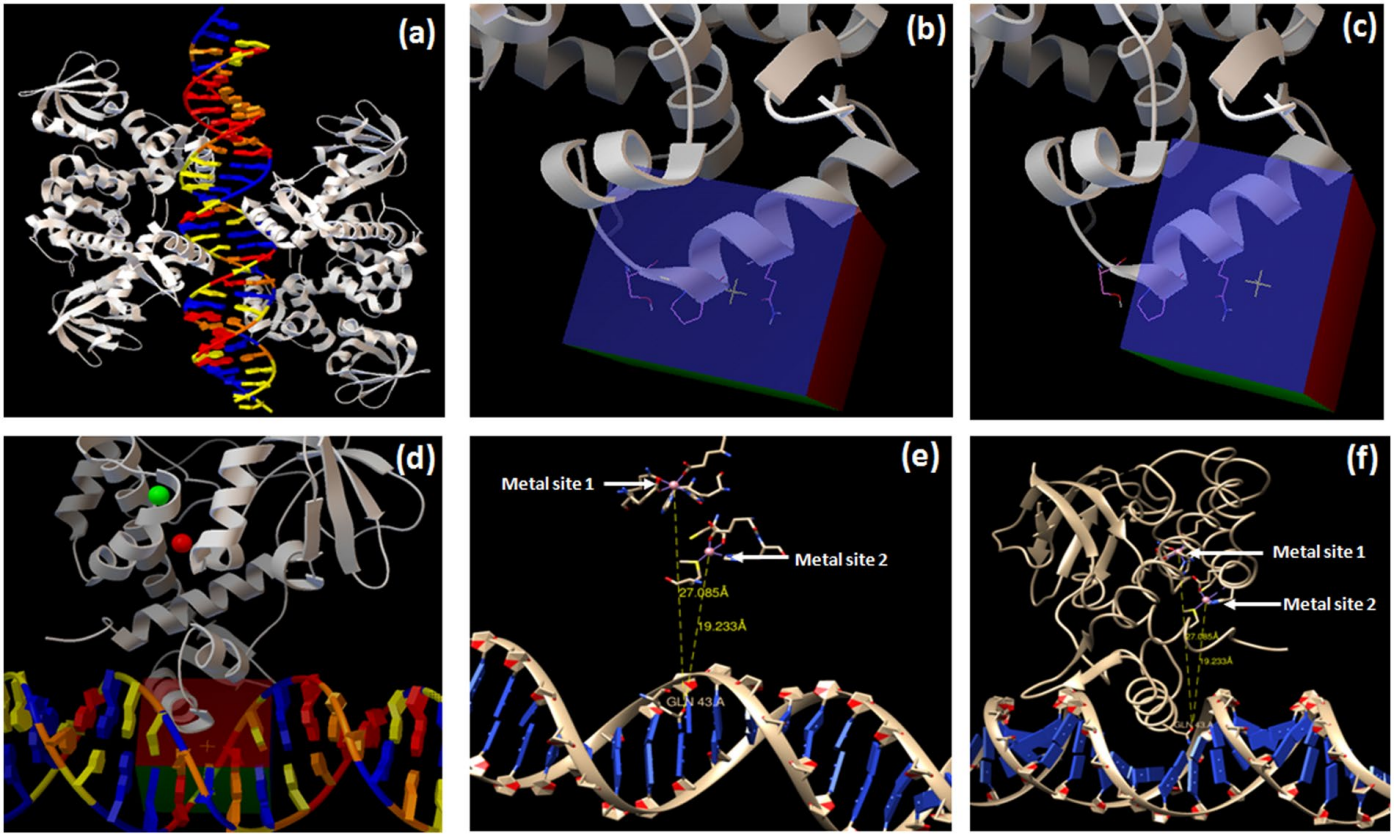
IdeR crystal structure and docking sites employed in this study. This figure depicts the spatial arrangement of IdeR, DNA and the docking sites. (**a**) IdeR homodimers bound to the cognate DNA sequence. (**b**) Docking site 1 at the DNA binding helix of IdeR. (**c**) Docking site 2 at the DNA binding helix of IdeR. (**d**) The spatial arrangement of docking site 1 with the metal binding site 1 (green) and site 2 (red) and cognate DNA sequence. (**e**) Distance between Gln 43 (present in the DNA binding helix) to the metal binding site 1 (27.085 Å) and site 2 (19.233 Å). It is evident from the distances depicted, the metal binding sites are far away from the DNA binding helix. (**f**) Distance between Gln 43 and metal binding sites along with the IdeR tertiary structure. (Images a to d were generated by using the software Autodock 4.2^25^ and e, f were generated by using the software chimera^43^).

In order to carry out the structure based inhibitor identification, we filtered the NCI library containing 260,071 compounds based on the Lipinski rule of five and drug likeness using the online FAF-server^24^ which resulted in 95,748 compounds (http://fafdrugs3.mti.univ-paris-diderot.fr/). These were subsequently employed for docking studies by using Autodock 4.2^25^. A number of IdeR monomer and DNA-bound crystal structures are available in PDB which provided key insights into the critical residues involved in the DNA binding which corroborate the results of footprinting experiments carried out by Gold *et al*.^16^. Figure 1a depicts the structure of IdeR homodimers bound to its cognate DNA molecule (PDB ID-1U8R). Based on the literature, Ser 37, Pro 39 and Gln 43 residues of the crystal structure of IdeR were identified to be important for binding to the DNA and were chosen as the grid center (docking site 1), which was used to dock the filtered NCI library (Fig. 1b). Based on the visual inspection of the interactions of the IdeR surface with DNA, a second docking site, adjacent to the docking site 1, was also selected with grid center at Ser 42 and Gln 43 (docking site 2) as depicted in Fig. 1c. It is to be noted that these two docking sites are present far apart from the metal binding sites (Fig. 1d–f). The relative spatial arrangement of the iron binding sites to the docking site 1 at the DNA binding helix is illustrated in Fig. 1d. Using these two sites, docking was performed and the compounds were shortlisted based on their free energies of binding. The average docking scores of the top scoring molecules from both the sites were in the range of −6 to −7 kcal/mol and are given in Table S1. Subsequently, a total of 123 compounds from site 1 and site 2 were procured from NCI and evaluated for their *in vitro* inhibitory potential to inhibit IdeR activity by employing EMSA.

### Inhibitory potential of the compounds against the DNA binding activity of IdeR

**Figure 2:**
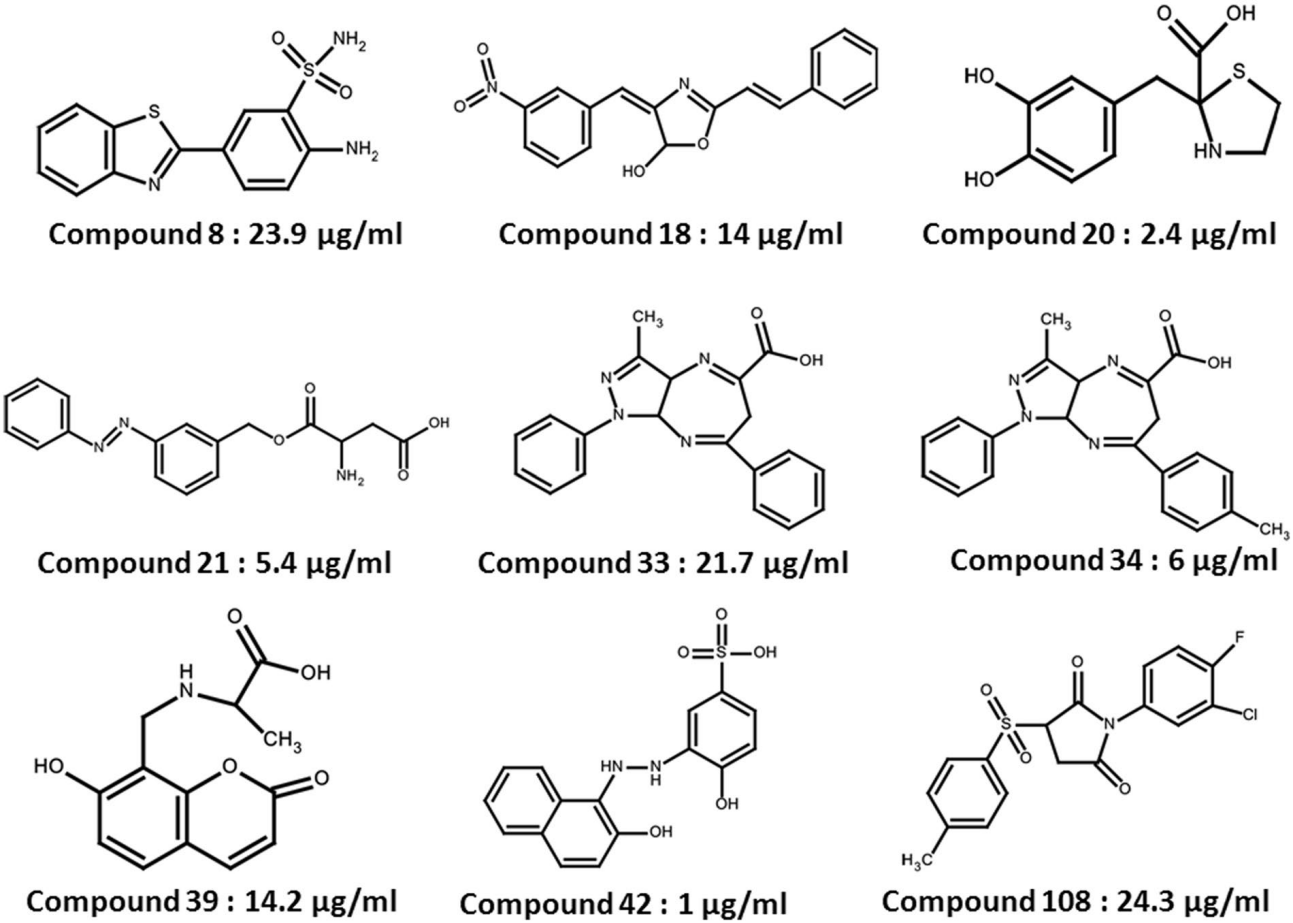
Structures of the most potent molecules and their respective IC_50_ values as determined by employing EMSA.

**Figure 3:**
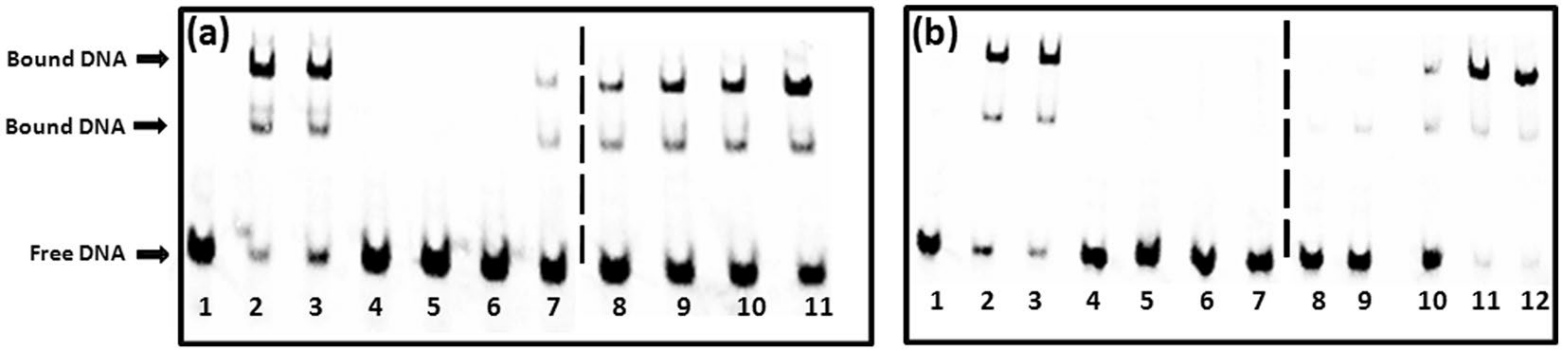
Dose response study of various molecules by employing EMSA. (**a**) Compound I-20, (**b**) Compound I-42. Dotted lines represent two different polyacrylamide gels. Lane 1-Free DNA, Lane 2-Protein+DNA, Lane 3-Protein+DNA+DMSO (−ve control), Lane 4–100 µg/ml, Lane 5–50 µg/ml, Lane 6–25 µg/ml, Lane 7–12.5 µg/ml, Lane 8–6.25 µg/ml, Lane 9–3.12 µg/ml, Lane 10–1.56 µg/ml, Lane 11–0.78 µg/ml, Lane 12–0.39 µg/ml (only in Fig. 3b). (For IC_50_ calculations, Protein+DNA and Protein+DNA+DMSO, control reactions were loaded in every gel and percentage of bound DNA for each inhibitor concentration was calculated by comparing the control reactions loaded in the same gel).

**Figure 4:**
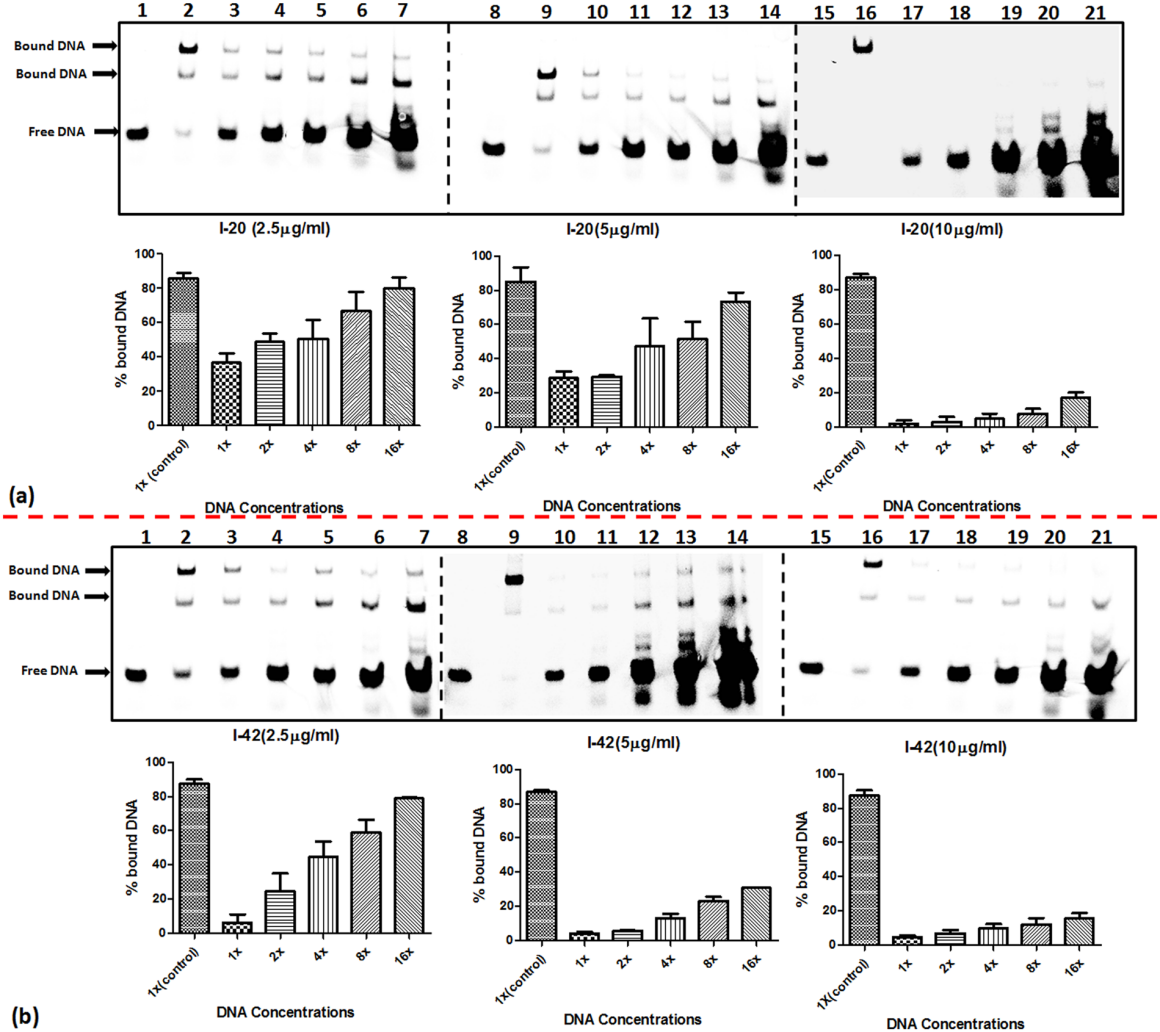
Competition experiments with compounds I-20 and I-42 and labeled DNA. Each gel depicts the alleviation of inhibition exhibited by compound I-20 (**a**) and compound I-42 (**b**) in the presence of increasing DNA. Lanes 1, 8, 15- free DNA (0.8 pmoles), lanes 2,9,16- IdeR+DNA (0.8 pmoles)+DMSO (vehicle control), lanes 3, 10, 17- IdeR+DNA (0.8 pmoles)+ inhibitor, lanes 4, 11, 18 - IdeR+ DNA (1.6 pmoles)+ inhibitor, lanes 5, 12, 19 - IdeR+DNA (3.2 pmoles)+ inhibitor, lanes 6, 13, 20 - IdeR+DNA (6.4 pmoles)+ inhibitor, lanes 7, 14, 21 - IdeR+DNA (12.8 pmoles)+ inhibitor. The bar diagram represents the percent bound DNA at varying DNA concentrations (x represents 0.8 pmoles of labeled DNA, dotted lines represent separate gels). The data is represented as Mean ± S.E.M by values obtained from two independent experiments.

**Figure 5:**
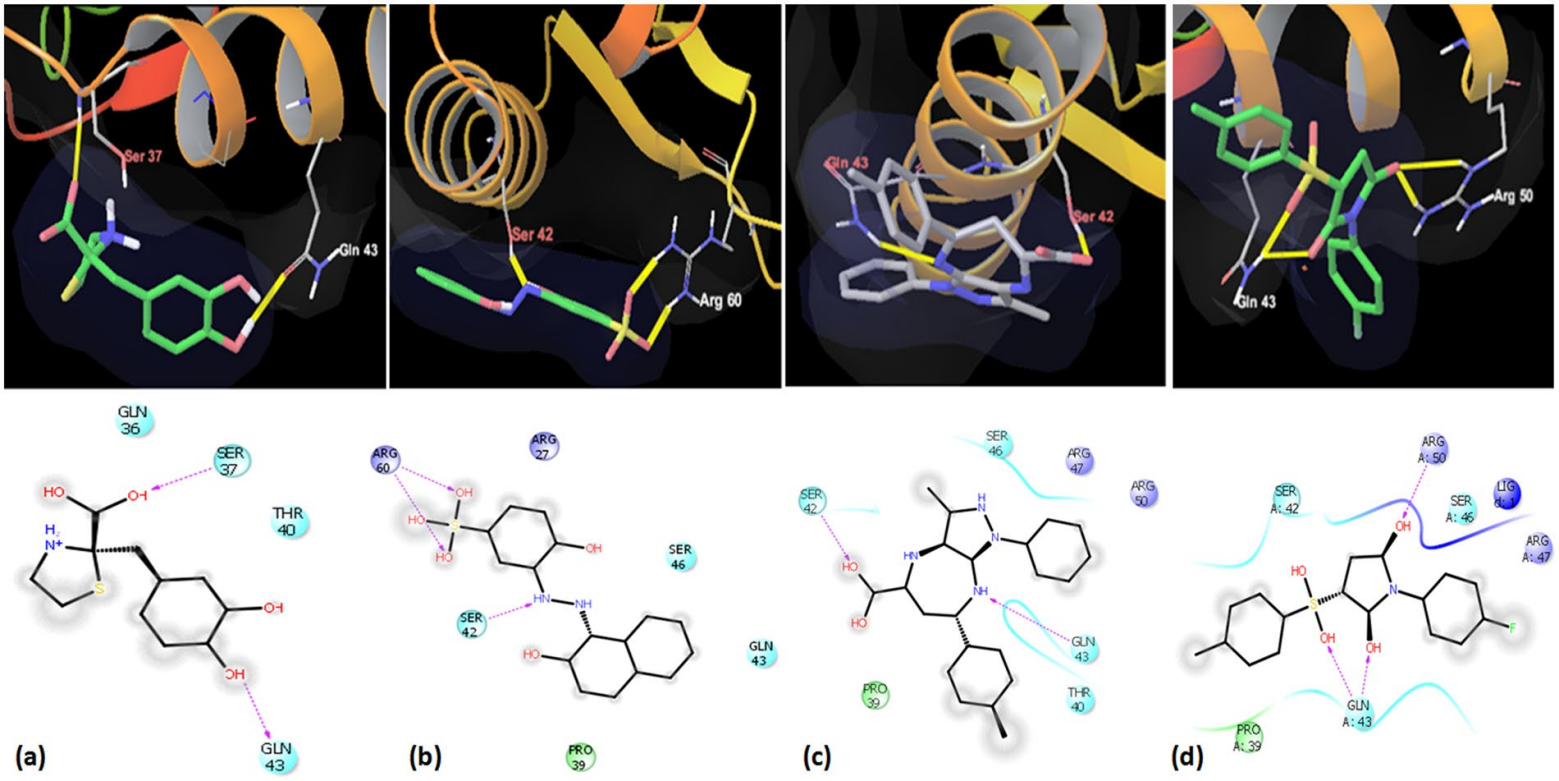
Binding mode analyses of (**a**) Inhibitor I-20- NSC 281033, (**b**) Inhibitor I-42-NSC 12453, (**c**) Inhibitor I-34- NSC 662444, (**d**) Inhibitor I-108- NSC 282699 (Yellow dash represents the Hydrogen bond between the ligand and protein residues).

EMSA was employed to evaluate the ability of the shortlisted compounds to inhibit the DNA binding activity of IdeR. For this, *ideR* gene was expressed and IdeR was purified to near homogeneity by Ni-NTA chromatography. One hundred twenty three compounds were screened at a fixed concentration of 100 µg/ml wherein 18 compounds exhibited more than 40% inhibition of the DNA binding activity of IdeR as given in Table S1. Subsequently, IC_50_ values for these 18 compounds were determined by employing varying concentrations of the compounds ranging from 0.4 µg/ml to 100 µg/ml. Nine compounds exhibited IC_50_ values less than 25 µg/ml, which were further selected for pharmacophore development and screening work, structures of these compounds are given in Fig. 2. (Prefix I is given to the compounds identified from this initial NCI library screening). The two most potent compounds, compound I-20 (NSC 281033) and I-42 (NSC 12453) exhibited IC_50_ values of 2.4 µg/ml (Fig. 3a) and 1 µg/ml (Fig. 3b), respectively. Further, we assessed the inhibition of the IdeR DNA binding activity of top 6 inhibitors (I-8, I-20, I-21, I-34, I-39 and I-42) exhibiting IC_50_ ≤ 25 µg/ml in the presence of increasing concentrations of the DNA (0.8 pmoles to 12.8 pmoles) and the results clearly demonstrate that all the 6 compounds inhibit the activity by competing with DNA (Fig. 4 and Fig. S1). The competition studies for compounds I-20 and I-42, as shown in Fig. 4, demonstrate that increasing the DNA concentration alleviates the inhibition exerted by the compounds as is evident by increase in the percentage of bound DNA (lanes 2–7 for 2.5 µg/ml, lanes 9–14 for 5 µg/ml and lanes 16–21 for 10 µg/ml). Moreover, this competition is dose dependent as it was observed that at any fixed DNA concentration, the per cent bound DNA decreases as the amount of inhibitor is increased. The competition results for the rest of the four compounds are depicted in Fig. S1. We also carried out experiments to assess the effect of Fe^2+^ instead of Ni^2+^ on the binding of IdeR to the DNA as well as the binding inhibition of the molecules as shown in Fig. S2. We found that 100 µM iron leads to equivalent binding as is seen in 200 µM Ni^2+^ (Fig. S2, lanes 2 and 3). However, further increase in the iron concentration hampers the DNA binding activity of IdeR (Fig. S2, lane 4). This could be due to the high redox instability of Fe^2+^ in *in vitro* conditions as already reported in the literature^26^. Further, it was observed that there was no effect of iron on the inhibitory potential of the compounds I-42, I-20 and I-34 (Fig. S2, lanes 5–7). Some of the compounds used in this study belong to metal chelators groups, however, our competition assays involving six compounds showed that all of them exerted the observed inhibition by competing with DNA substrate. The docking studies indicated the interactions of compounds I-20 and I-42 with various residues present at the DNA binding helix. The predicted key interactions made by compound I-20 having a thiazolidine-benzyl scaffold include hydrogen bond between the hydroxyl group at the 3^rd^ position of the benzyl ring with the carboxyl group of Gln 43 and the oxygen in the carboxylic acid attached to the thiazolidine ring interacts with the amino group of Ser 37 (Fig. 5a). The diazene group of compound I-42 which bears a benzyl-naphthalenyl scaffold is predicted to form a hydrogen bond with the hydroxyl group of Ser 42 and the oxygen atoms in the sulfonic acid is predicted to form a network of hydrogen bonding with the two amino groups of the side chain of Arg 60 (Fig. 5b). This provides key insights into a few other novel critical residues (apart from Ser 37, Pro 39 and Gln 43), that can be exploited while carrying out the structure activity relationship studies for inhibitor identification against IdeR. Compounds I-33 and I-34 containing a common dihydropyrazolo-diazepene scaffold exhibited an IC_50_ of 21.7 µg/ml and 6.1 µg/ml, respectively. The predicted key interactions made by compound I-34 include a hydrogen bond between nitrogen of the diazepine moiety with the amino group of Gln 43 and the oxygen atom of the carboxyl group attached to the diazepine moiety with the hydroxyl group of Ser 42 (Fig. 5c). Inhibitor I-108 exhibited an IC_50_ of 24 µg/ml and its predicted key interactions include formation of a network of hydrogen bond between oxygen atom of sulfonyl and pyrrolidine ring with amino group of Gln 43 and between a second oxygen atom of pyrrolidine ring with Arg 50 (Fig. 5d). Thus, the initial docking followed by EMSA provided few potent inhibitory molecules with IC_50_ ≤ 25 µg/ml and which make key interactions with the various residues of IdeR involved in its DNA binding activity.

### Energy based Pharmacophore Generation, Docking and EMSA inhibition study

**Figure 6:**
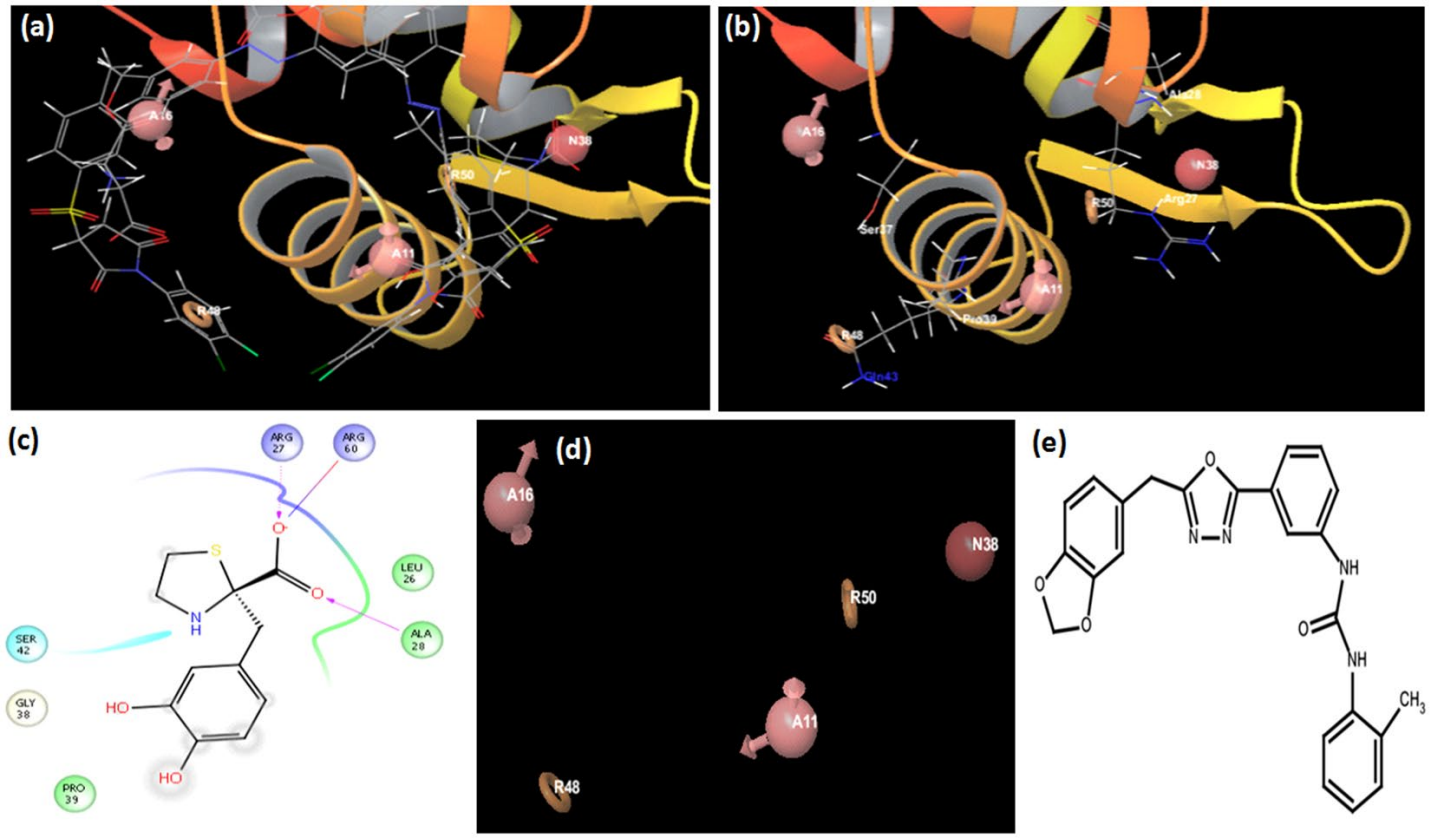
Energy Based Pharmacophore development. (**a**) Multiple conformations of I-20 (IC_50_-1 µg/ml) bound to the DNA binding surface of IdeR. (**b**) Various pharmacophore points in proximity with Ser 37, Pro 39, Gln 43, Arg 27 and Ala 28. (**c**) 2-D Interaction map of I-20 with various residues of the DNA binding surface of IdeR. (**d**) Final 5-Point pharmacophore taken for subsequent screening of the drug like database of ZINC. (A16 and A11 refers to Acceptor feature, R48 and R50 refers to Aromatic ring feature and N38 refers to negative ionizable group). (**e**) 2-D structure of IA-8 3-[3-[5-(1, 3-benzodioxol-5-ylmethyl)-1, 3, 4-oxadiazol-2-yl]phenyl]-1-(o-tolyl) urea with an IC_50_ of 60 µg/ml.

To provide key insights into the critical features required for inhibition of DNA binding ability of IdeR, an energy based pharmacophore model was developed by using e-pharmacophore script^27, 28^ available in the Schrodinger Software package. An energy based pharmacophore extracts and weighs interactions made by an inhibitor with the protein by using the descriptor information generated by the docking performed through Glide^29–31^ and provides a map of critical features contributing to the inhibitor-protein interaction. All the nine compounds (IC_50_ < 25 µg/ml) were docked at site 1 again by using Glide in the Schrodinger package. However, the docking scores of all the molecules were in the range of −1 to −2 kcal/mol as compared to −6 to −7 kcal/mol generated by Autodock 4.2^25^, except compound I-20 which exhibited a score of −4.25 kcal/mol. Subsequently, the pharmacophore hypothesis was generated on the basis of the XP descriptor file generated by docking the compound I-20 by using Glide. This file was fed into the e-pharmacophore script and a pharmacophore model consisting of 5 features was generated which comprised of two hydrogen bond acceptors, two rings and one negative ionizable group. Multiple conformations of compound I-20 provided a map of its possible key interactions with the residues present on the DNA binding groove of IdeR as shown in Fig. 6a. The selection of each pharmacophore point was made on the basis of their proximity with the key residues involved in DNA binding i.e Hydrogen Bond Acceptor (A16) with Ser 37, Aromatic Ring (R48) with Gln 43, Hydrogen Bond Acceptor (A11) with Pro 39 (Fig. 6b). Two additional points were included namely Aromatic ring (R50) and Negative ionizable group (N38) which are shown to be interacting with Ala 28, Arg 60 and Arg 27 as shown in (Fig. 6c). The final pharmacophore model (Fig. 6d) was used to screen the drug like database of ZINC consisting of ~13 million compounds by using Phase module in Schrodinger package^32, 33^. This resulted in ~110,000 molecules with a filter of 4 out of 5 molecular features to be matched. 2-D and 3-D descriptors of these hits were generated by qikprop module in Schrodinger, which were subsequently used to screen these molecules on the basis of mycobacterial cell wall permeability by using MycPermCheck^34^ (http://www.mycpermcheck.aksotriffer.pharmazie.uni-wuerzburg.de/) online server which resulted in ~39,000 molecules. These molecules were docked at site 1 by employing Autodock 4.2^25^ resulting in 35 molecules with free energy binding scores above −7 kcal/mol which were further shortlisted on the basis of their structural diversity by using tanimoto filter of 0.8 using the software Openbabel^35^. Subsequently, 22 molecules were selected, procured and tested for their *in vitro* inhibitory potential by employing EMSA out of which compound IA8 (ZINC 21711688, Ambinter ID, 20476786, prefix IA is given to this compound procured from Ambinter, which is identified in the pharmacophore based screening) (Fig. 6e) exhibited an IC_50_ of 61.3 µg/ml. A probable reason for the identification of poor inhibitors by this approach could be the chemical composition of the library screened. The nature of the library employed for pharmacophore and docking studies ultimately reflects upon the quality and potency of the inhibitors identified, however, a few diverse libraries are generally required to be screened for this purpose and indeed the parameters for selection of a library that will result into potent hits are difficult to decide a priori. Another probable reason for a low hit rate and weak IC_50_ value could be a low docking score (−4.25 kcal/mol) of compound I-20 on the basis of which the model was developed. Thus, the docking may not be able to completely capture the interactions that could corroborate with a potent IC_50_ value of 2.4 µg/ml. However, to the best of our knowledge, there is no published report of IdeR inhibitors and co-crystallized structures of inhibitors with IdeR which could have provided substantial information about the native conformation and key interactions of inhibitor bound with the protein. Such information has been the basis for developing energy based pharmacophore model in numerous studies^36–39^. Thus, this study presents an *in vitro* validated five point pharmacophore model which provides insights into the features required for IdeR inhibition and can be further optimized and used for future virtual screening endeavors.

### Structure similarity based search for analogs of the potent IdeR lead molecules

**Figure 7:**
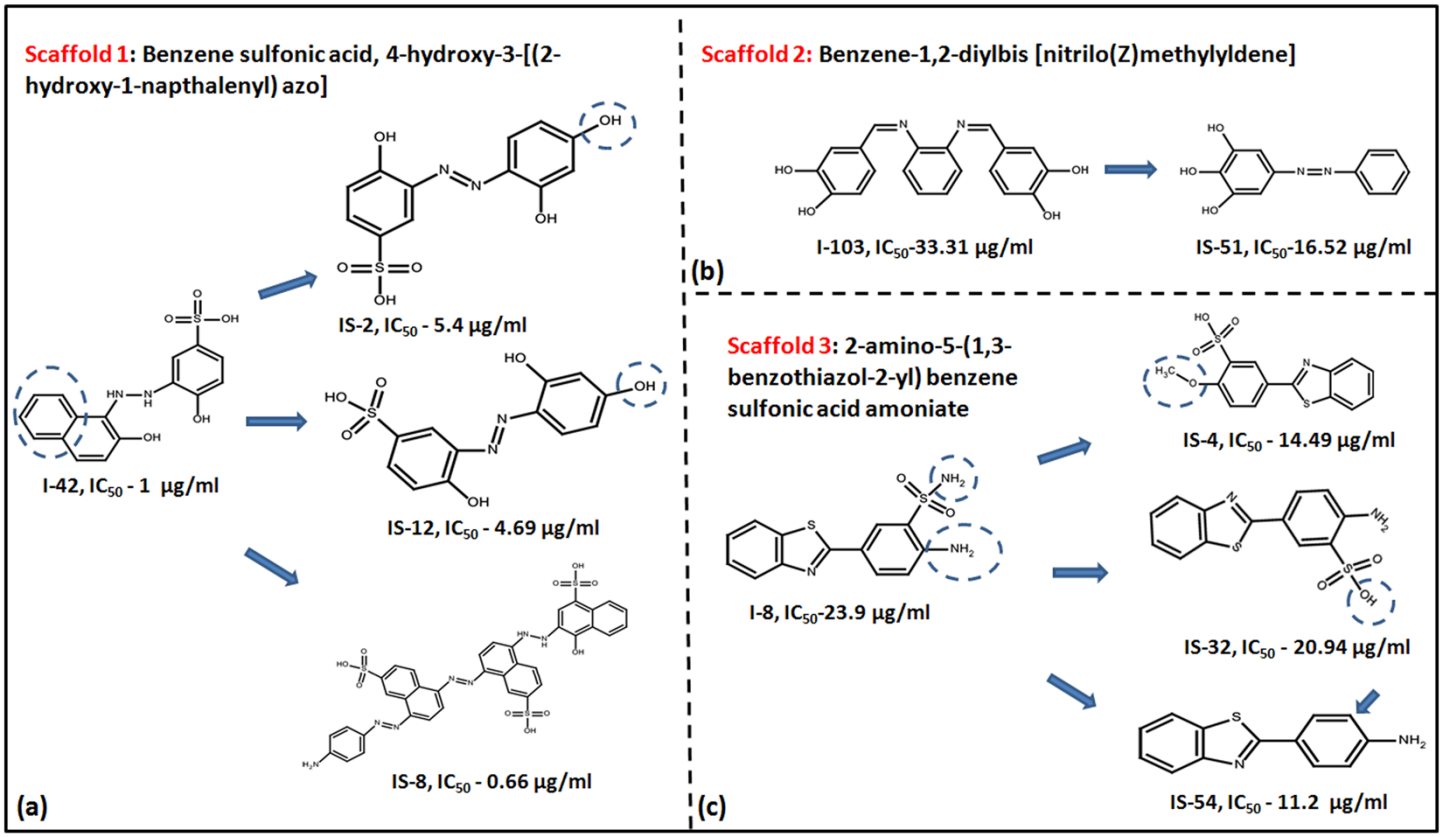
Structure similarity based identification of three potent scaffolds. This figure represents three scaffolds identified by testing analogs of compounds I-42 (**a**), I-103 (**b**) and I-8 (**c**) and their corresponding IC_50_ values.

In order to identify more potent analogs of the compounds identified in the initial inhibitory screening, a structural similarity based screening of NCI library was carried out. For this,10 molecules namely (I-8, I-18, I-20, I-33, I-34, I-39, I-42, I-85, I-103, I-108) were taken as query molecules individually and the complete NCI database consisting of ~3,00,000 compounds was searched based on the 2-D fingerprint similarity as measured by the Tanimoto coefficient with a cut-off of 0.7 using the software Openbabel and similar analogs were shortlisted. In total, 66 molecules were procured from NCI and tested for their potential to inhibit DNA binding ability of IdeR by employing EMSA. Out of which, 17 molecules exhibited more than 40% inhibition at 100 µg/ml and were shortlisted for the determination of their IC_50_ values as given in Table S2. (Prefix IS is given to the similar analogs identified from this structure similarity based screening approach). Nine out of 16 molecules belonging to the class of compound 42 exhibited potent inhibition with IC_50_ of five molecules ≤ 15 µg/ml thereby establishing benzene sulfonic acid,4-hydroxy-3-[(2-hydroxy-1-naphthalenyl) azo] as a potent inhibitory scaffold and also provided insight into the key structural requirements for the inhibition of IdeR DNA binding ability. For eg, compound IS-2 and IS-12 bear a hydroxyl group at the position opposite to diazene moiety as compared to a phenyl ring in I-42 which emphasizes the fact that a bulkier aromatic group is required with a phenyl moiety as compared to a hydroxyl group which leads to a slight decrease in the potency as shown in Fig. 7a. Also, compound IS-8 with an IC_50_ of 0.66 µg/ml, essentially contains three repeating naphthalene azo sulfonic acid scaffold thereby highlighting the importance of this scaffold for IdeR inhibition. Compound IS-51 with an IC_50_ value of 16.5 µg/ml which is an analog of compound I-103 (IC_50_ of 33.3 µg/ml) exhibited a two-fold increase in potency as depicted in Fig. 7b. The presence of an extra diol-phenyl group in the core scaffold, which is present in the lead molecule I-103 is predicted to hinder the binding of the inhibitor with the protein as its absence in IS-51, results in better inhibition. Analogs of compound I-8 (IC_50–23.9_ µg/ml), also exhibited improved IC_50_ values with 14.49 µg/ml of IS-4, 20.94 µg/ml of IS-32 and a two fold increase in potency of IS-54, IC_50_ of 11.2 µg/ml as shown in Fig. 7c thereby suggesting a benzo-thiazol benzene sulfonic acid as another scaffold important for IdeR inhibition. This study thus made an attempt towards establishing three distinct scaffolds that are crucial for inhibiting the DNA binding activity of IdeR and can serve as a robust platform for the generation of more rationally designed analogs.

### Inhibition of *M. tuberculosis* in broth culture by using Resazurin Microtiter Assay (REMA)

All the molecules that inhibited the IdeR DNA binding activity in EMSA were tested for their ability to inhibit growth of *M. tuberculosis* in broth culture by using Resazurin Microtiter Assay (REMA)^40, 41^. Resazurin oxidation-reduction based method provides visible distinction as well as fluorescence based measurement of the viability of the cells. To evaluate the MIC_90_ of the compounds inhibiting the growth of *M. tuberculosis* in broth culture, serial dilutions of the compounds were employed with *M. tuberculosis* cells growing in MB7H9 medium. Compound I-108, which exhibited an IC_50_ of 24.3 µg/ml, also exhibited an MIC_90_ of 17.5 µg/ml against *M. tuberculosis* as shown in Table 1. Compounds I-33 and I-34 exhibiting an IC_50_ of 21.7 µg/ml and 6.1 µg/ml inhibited the growth of *M. tuberculosis* at 93.3 µg/ml and 86.6 µg/ml, respectively (Table 1). Also, compounds IS-4 and IS-54 (analogs of compound 8) exhibited an MIC_90_ of 100 µg/ml and 84.8 µg/ml respectively, although its parent analog, compound I-8 exhibited no inhibition of *M. tuberculosis* growth in MB7H9 medium (Table 1). It is to be noted that further investigations are required to correlate the whole cell inhibition exhibited by the five compounds with the inhibition of IdeR inside the cells, and the possibility of off-target effects cannot be negated.

**Table 1:** Inhibitors exhibiting whole cell *M. tuberculosis* broth inhibition and cytotoxicity studies.

Compound No.	NSC ID	MIC_90_ of compounds against *M. tuberculosis* (µg/ml)	Inhibition in THP1 (IC_50_ in µg/ml)	Inhibition in HEK (IC_50_ in µg/ml)	Inhibition in MDCK (IC_50_ in µg/ml)	Inhibition in HepG2 (IC_50_ in µg/ml)
I-33	662443	93.3 ± 8.16	>200	>200	>200	>200
I-34	662444	86.6 ± 7.8	>200	>200	>200	>200
I-108	282699	17.5 ± 3.06	167.5 ± 5.6	>200	>200	>200
IS-4	33755	100	165.8 ± 3.72	164.7 ± 1.24	172.8 ± 2.3	176.3 ± 1.6
IS-54	34445	84.8 ± 8	181.6 ± 5.2	161.1 ± 4.22	174.3 ± 6.4	167.4 ± 3.78
Rifampicin		0.05–0.2	>100	>100	>100	>100

### Cytotoxicity studies against mammalian cell lines

Since, *M. tuberculosis* primarily resides inside the host macrophages and in order for a compound to exhibit a good ADME/toxicity profile, it is important for it to be non-cytotoxic against hepatic and kidney cells of the host, thus, we analyzed the cytotoxicity of the compounds against THP-1 (human monocytic macrophage cell line), HepG2 (human liver cancer cell line), MDCK (Madine Darby Canine Kidney cell line) and HEK (Human Embryonic Kidney cell line) respectively. Compounds I-33 and I-34 exhibited no cytotoxicity up to 200 µg/ml (the maximum concentration tested) in all the four cell lines tested whereas compound I-108 exhibited no cytotoxicity in three of the four cell lines tested with slight toxicity in THP-1 cells. However, IS-4 and IS-54 exhibited slight toxicity in all the four cell lines tested, as given by their respective IC_50_ values shown in Table 1. Hence, I-33, I-34 and I-108 were further selected to evaluate their inhibitory potential against the growth of *M. tuberculosis* residing in macrophages.

### Evaluation of the compounds to inhibit the intracellular *M. tuberculosis*

**Figure 8:**
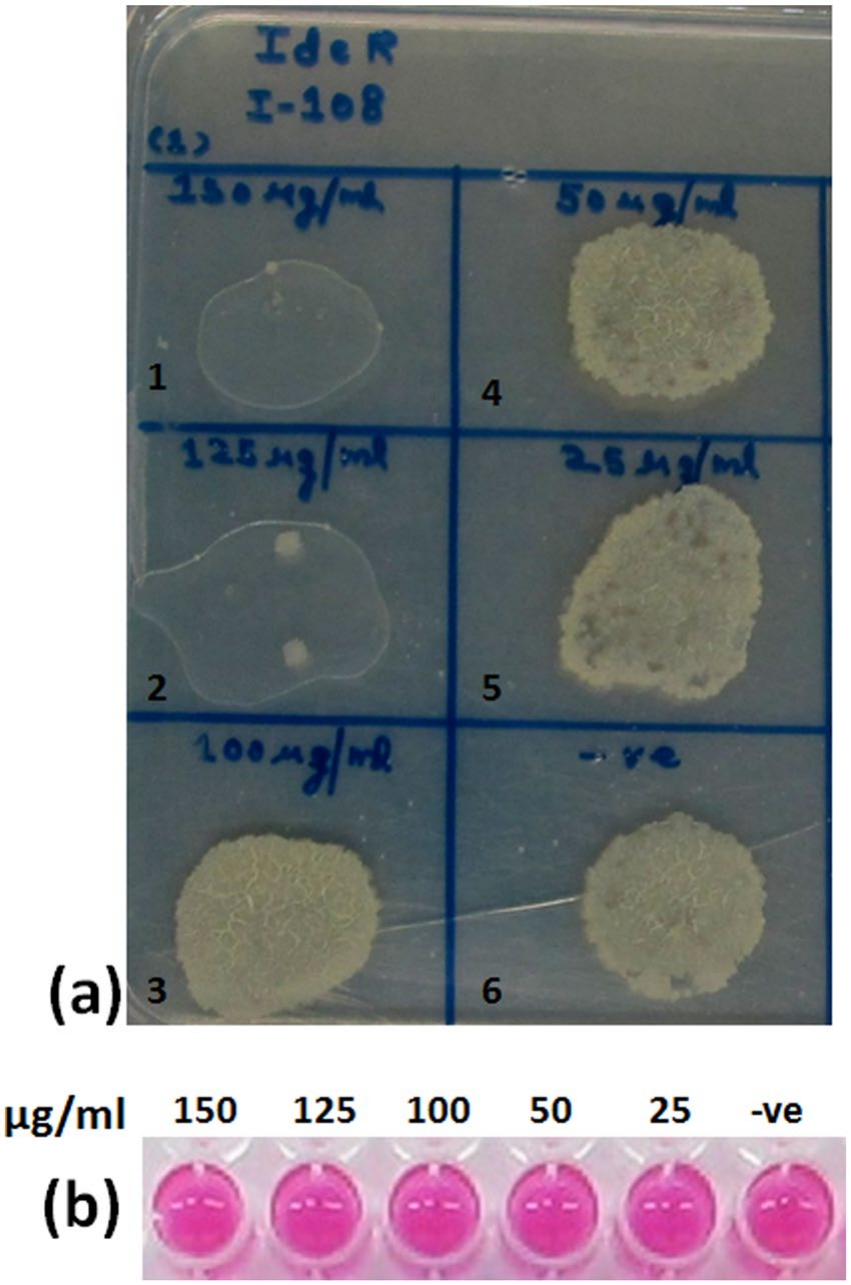
Evaluation of inhibitory potential of compound I-108 against the growth of intracellular *M. tuberculosis*. (**a**) The influence of varying concentrations of I-108 on the growth of intracellular *M. tuberculosis* as analyzed on the agar plates. Panel a depicts the emergence of pin point colonies at the concentrations of 150 μg/ml and 125 μg/ml as compared to prominent growth of bacilli at lower concentrations of I-108. (**b**) Alamar blue assay to measure the viability of uninfected THP-1 cells in the presence of varying concentrations of I-108. The upper lane of panel b depicts the various concentrations of I-108 employed for the alamar blue assay (in μg/ml). Lanes 1–6 in panel a depicts following concentrations of I-108: Lane 1–150 μg/ml, Lane 2–125 μg/ml, Lane 3–100 μg/ml, Lane 4–50 μg/ml, Lane 5–25 μg/ml, Lane 6- -ve (only cells).

Based on the inhibitory potential of the compounds against the activity of IdeR, growth inhibition of *M. tuberculosis* in broth culture as well as evaluation of cytotoxicity in various mammalian cell lines, we shortlisted three molecules I-33, I-34 and I-108 with MIC_90_ values of 93 μg/ml, 86 μg/ml and 17.5 μg/ml, respectively, for the evaluation of the ability of these compounds to inhibit the growth of *M. tuberculosis* inside macrophages. PMA activated THP-1 cells were infected with *M. tuberculosis* at an MOI of 1:5 and were incubated in the presence of varying concentrations of the compounds (I-108–25 μg/ml to 150 μg/ml, I-33 and I-34–100 μg/ml to 500 μg/ml). The growth of the intracellular bacteria was analyzed on the agar plates. It was observed that in the presence of I-108, only a few pin pointed colonies could be seen at the concentrations of 150 μg/ml and 125 μg/ml, however, prominent growth of bacilli was seen at lower concentrations demonstrating that I-108 is able to limit the growth of intracellular bacteria, albeit, at the high concentrations of 150 μg/ml and 125 μg/ml. Importantly, I-108 is not toxic to the THP-1 cells at these concentrations, as evaluated by alamar blue assay (Fig. 8). Also, concentrations higher than 150 μg/ml were not included due to its toxicity to the THP-1 cells at higher concentrations. It is to be noted that the highest concentration employed for I-108 of 150 μg/ml is quite high for inhibition studies and any molecule inhibiting the growth of intracellular bacteria in this higher range is regarded only as a weak inhibitor. Therefore, inhibition exhibited by I-108 at higher concentrations of 150 μg/ml and 125 μg/ml should be considered only as a proof of concept and more rational modifications of this compound are required to improve its potency. Compounds I-33 and I-34 with MIC_90_ value of 93 μg/ml and 86 μg/ml, respectively, exhibited negligible inhibition of the growth of the intracellular bacteria as numerous small colonies were seen at 500 μg/ml (highest concentration tested) in the case of both I-33 and I-34, although, they were non-toxic to the THP-1 cells at this high concentration of 500 μg/ml, while at lower concentrations, the growth of the intracellular bacteria was unaffected (Fig. S3). Thus, I-33 and I-34 having weak MIC_90_ values of 93 μg/ml and 86 μg/ml, respectively, were unable to inhibit the growth of *M. tuberculosis* even at a very high concentration of 500 μg/ml. Rifampicin was used as a positive control. One of the possible reasons for the inability of I-33 and I-34 to inhibit the growth of intracellular bacteria as well as exert no cytotoxicity could be their poor permeability in the mammalian cells. Also, the determination of the inhibitory potential of these compounds by spotting on the agar plates is a qualitative but a relatively high throughput method and more detailed studies such as enumeration by CFU are required to determine the precise MIC_99_ values. However, these studies are quite labor extensive and thus, studies focused at first improving their inhibitory potential which would result in more potent analogs are warranted before undergoing such labor extensive studies. Thus, our study provides one molecule, I-108, which exhibited inhibitory potential against the activity of IdeR, growth inhibition of *M. tuberculosis in vitro*, low toxicity against various mammalian cell lines and also a moderate inhibition of growth of intracellular bacteria. Further optimization studies of this lead molecule can result into more potent compounds.

## Conclusion

*M. tuberculosis* IdeR has remained an attractive drug target for almost a decade. Various genetic studies pertaining to its *in vitro* and *in vivo* essentiality, biochemical studies which include the characterization of IdeR such as crystal structure determination and iron and DNA binding kinetics, have laid a robust platform to carry out structure based drug discovery against IdeR. Our study involved the virtual screening followed by testing of 123 compounds *in vitro* by employing EMSA, which yielded few potent inhibitory compounds with IC_50_ ≤ 25 µg/ml. Subsequent energy based pharmacophore modeling followed by docking studies led to the identification of a five point pharmacophore model that provided key insights into the features responsible for IdeR inhibition and can be further optimized to carry out more virtual screening studies. A parallel structure similarity based approach led to the identification of a benzene sulfonic acid, 4-hydroxy-3-[(2-hydroxy-1-naphthalenyl) azo] scaffold and 2-amino-5-(1,3- benzothiazol-2-yl) benzene sulfonic acid class of compounds inhibiting the DNA binding activity of IdeR. The MIC and cytotoxicity studies provided few compounds such as I-108 that exhibited IC_50_ of 24.3 µg/ml, MIC_90_ of 17.5 µg/ml, negligible cytotoxicity in three of the four mammalian cell lines and was also able to limit the growth of intracellular bacteria, albeit at a high concentration of 125 µg/ml. Also, compound I-34 exhibited IC_50_ of 6 µg/ml, moderate MIC_90_ values of 86.6 µg/ml, negligible toxicity in various mammalian cell lines but was unable to limit the growth of intracellular bacteria even at a very high concentration of 500 µg/ml. Thus, I-108 can serve as a starting point for carrying out more rational modifications and structure activity relationship studies.

**Figure 9:**
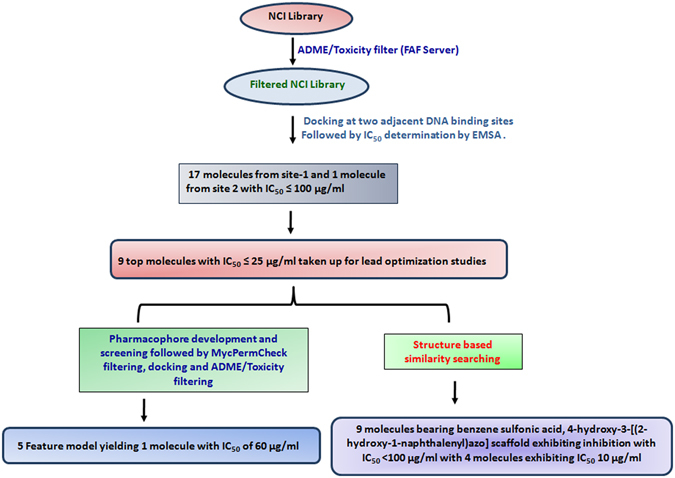
Flowchart of the strategy followed in this work.

This study opens avenues for various studies related to the drug discovery efforts against Iron Dependent Regulator, an essential gene of *M. tuberculosis*. In our study, we identified molecules inhibitory to IdeR activity which is useful for further studies. Determination of IdeR co-crystallized structures bound to these inhibitors would provide information about the important residues crucial for determining the DNA binding activity of IdeR which in turn would help in the development of more potent inhibitors. Moreover, the molecules identified in this study can serve as a robust platform for carrying out more rational modification and structure activity relationship studies. Some of the compounds used in this study belong to metal chelators groups, however, our competition assays involving six compounds showed that all of them exerted the observed inhibition by competing with DNA substrate. Thus, our study follows a combination of various *in silico* approaches well supported and validated by subsequent *in vitro* experiments (pipeline depicted in Fig. 9) to identify various lead molecules against IdeR, an important drug target of *M. tuberculosis*.

## Materials and Methods

### Library generation and docking studies

Three dimensional structures of molecules belonging to the Open NCI Database consisting of 260,071 compounds were downloaded from the NCI website (http://cactus.nci.nih.gov/download/nci/). The downloaded.sdf files were converted into.smi format by Openbabel^35^ software and shortlisted based on the Lipinski Rule and ADME/Toxicity parameters by using FAF-Drugs server^24^ (http://fafdrugs3.mti.univ-paris-diderot.fr/). This shortlisted library was employed for the subsequent virtual screening studies. The three-dimensional crystal structure of IdeR (1U8R) was downloaded from the PDB database (http://www.rcsb.org/pdb/home/home.do) and Autodock 4.2^25^ was used to perform the docking calculations. The structure was prepared by deleting the water molecules followed by the addition of the hydrogen atoms and the protein was saved in.pdbqt format. Genetic algorithm with default parameters was employed for the docking calculations. All the calculations were carried out on 48 node cluster of X2200 Sun Fire with dual core AMD processor with CENT-OS version 5. The compounds were procured from the Drug Synthesis and Chemistry Branch, Developmental Therapeutics Program, Division of Cancer Treatment and Diagnosis, National Cancer Institute, National Institutes of Health, Bethesda, MD, USA.

### Cloning of the gene, expression and purification of IdeR

The *M. tuberculosis ideR* gene was PCR amplified by using *M. tuberculosis* genomic DNA as the template. The sequence-5′gataatggtaccactagtcatatgaacgagttggttgatacc 3′ with *Nde*I restriction site at 5′ end was used as the forward primer and sequence-5′gataataagcttattatttttcgaactgcgggtggctccaagcgcttcagactttctcgaccttgac 3′ with *Hind*III restriction site was used in the reverse primer. The reverse primer also contained streptactin tag for purification. The amplified product was digested with *Nde*I and *Hind*III and cloned into pET28c digested with the same enzymes resulting in the construct pET28c/*IdeR*, which was subsequently used for carrying out the expression and purification studies. BL21 (λDE3) cells transformed with pET28c/*IdeR* were grown to an A_600nm_ of 0.8–1.0 in LB media at 37 °C containing 25 µg/ml of kanamycin and synthesis of IdeR protein was induced by the addition of 1 mM isopropyl-1-thio-β-D-galactopyranoside (IPTG) at 37 °C. After induction for 3 hours, the *E. coli* cells were harvested by centrifugation at 4 °C, 6340 g for 10 minutes and resuspended in 30 ml lysis buffer (20 mM Tris-HCl (pH 8.0), 50 mM NaCl, 1 mM phenylmethylsulfonylfluoride (PMSF), 2 mM β-mercaptoethanol, and 5 mM Imidazole). The resuspended cells were sonicated and the lysate was centrifuged at 12,000 g for 45 minutes at 4 °C and the supernatant was incubated with 1.5 ml Ni-NTA resin pre-equilibrated with buffer containing 20 mM Tris-HCl (pH 8.0), 50 mM NaCl and 5 mM imidazole for 1 hour. The flowthrough was collected and the resin was washed twice with 20 ml each of buffers (20 mM Tris-HCl (pH8.0), 50 mM NaCl) containing 5 mM and 10 mM imidazole and protein was eluted as 1 ml fractions by using the elution buffer (20 mM Tris-HCl, 50 mM NaCl and 250 mM imidazole). Protein estimation of the eluted fractions was carried out by Bradford assay, analyzed by SDS-PAGE on a 12.5% gel and samples were pooled and dialyzed against the buffer (20 mM Tris, 50 mM NaCl and 1 mM DTT) and concentrated by using an Amicon Ultra protein concentrator (Millipore, Billerica, MA, USA) with a 5 kDa filter and the purified protein was stored in aliquots at −80 °C.

### Electrophoretic Mobility Shift Assay (EMSA)

A synthetic double stranded oligonucleotide belonging to intergenic region between *MbtA* and *MbtB* containing IdeR binding region with sequence-5′ccctgttagcacaggctgccctaattttagtg3′ (sense strand) and Cy5 fluorophore label at 5′ end of both the strands was procured from Integrated DNA Technologies, Coralville, Iowa, U.S.A and used as a substrate for the assay. Binding reactions included 20 mM Tris-HCl at pH 8.0, 1 mM DTT, 50 mM KCl, 5 mM MgCl_2_, 200 µM NiSO_4_, 0.05 mg ml^−1^ poly (dI-dC), 0.05 mg ml^−1^ BSA, 10% glycerol as reaction buffer and purified IdeR (0.35 µg) in a reaction volume of 20 µl and the reaction was carried out at room temperature for 30 minutes. Samples were electrophoresed on a 6% polyacrylamide gel containing 40 mM Tris-acetate (pH-8.0), 10% glycerol, 200 µM NiSO_4_ and the running buffer (Tris-acetate-40 mM and 200 µM NiSO_4_). Gels were run at 90 V at room temperature and the bands were visualized by using phosphorimager (FLA-9000, Fujifilm Corporation, Minato-ku, Tokyo, Japan) with Cy5 filter and each band intensity was quantified by using the Multi Gauge software (FujiFilm Corporation, Minato-ku, Tokyo, Japan).

### *In vitro* Inhibition Assays

Compounds were dissolved in DMSO at a concentration of 5 mg/ml and screened at a fixed concentration of 100 µg/ml. Each reaction mix contained 0.35 µg IdeR and 0.4 µl of 5 mg/ml compound (final concentration of 100 µg/ml) in the reaction volume of 20 µl containing the reaction buffer with DMSO as the negative control. The reaction mix was incubated for 15 minutes at room temperature, followed by the addition of 0.8 picomoles of labeled DNA and further incubation was carried out for 30 minutes at room temperature. Subsequently, the reaction mix was loaded onto 6% polyacrylamide gel, electrophoresed and scanned by using FLA 9000. The molecules exhibiting more than 40% inhibition were further subjected to dose response studies with varying inhibitor concentrations ranging from 0.4 µg/ml to 100 µg/ml for the determination of their IC_50_ values. The concentration of the inhibitor resulting in 50% reduction in the bound DNA band was considered as the IC_50_ of that molecule and was calculated by using the following equation 1:

1 $$Inhibition\,( \% )=(1-\frac{Fluorescence\,unit,compound}{Fluorescence\,unit,control})\times 100$$

IC_50_ values were determined from the results of at least two independent tests and calculated from the inhibition curve using GraphPad Prism 5.

For competition experiments, % bound DNA was calculated by using the following equation 2:

2 $$Bound\,DNA\,( \% )=(\frac{Fluorescence\,unit,bound\,DNA\,bands}{Fluorescence\,unit,free\,DNA\,\,band})\times 100$$

Per cent bound DNA were calculated by using the results of atleast two independent tests and calculated from the One way ANOVA test by using the column bar graph of GraphPad Prism 5. The data is represented as Mean ± S.E.M. by values obtained from two independent experiments.

### For lead Optimization studies

#### Strategy 1 (a): Energy Based Pharmacophore Development

Five point energy based pharmacophore model was developed by using the e-pharmacophore^27, 28^ script available with Schrodinger Package (Schrodinger, LLC, New York, NY) by using the standard pharmacophore features namely hydrogen bond acceptor (A), hydrogen bond donor (D), hydrophobic group (H), negatively ionizable (N), positive ionizable (P) and aromatic ring (R). Energy minimized conformers with added hydrogens and stereoisomers were generated for the selected molecules by using LigPrep 2.5 module from Schrodinger package. These were docked at the active site of the protein by using Glide^29–31^ and an XP-descriptor file was generated which facilitated the generation of five-point pharmacophore. Subsequently, screening of ZINC drug like database was carried out by using Phase^32, 33^ (Phase, version 3.3, Schrodinger, LLC, New York, NY, 2011). Default settings were kept in place which included the generation of conformers and thorough sampling. Four out of 5 site points to be matched was set as criteria for filtering. All the calculations were carried out on an 8 node SunFire X2200 cluster with CENT-OS operating system.

#### 1(b): Generation of the Molecular Descriptors through Qikprop

The hits retrieved from the energy based pharmacophore were subsequently utilized for the generation of 2-D and 3-D descriptors which included their topological as well as ADME/Toxicity based descriptors. Qikprop module available in the Schrodinger Suite was employed for this purpose.

#### 1(c): Mycobacterium Cell wall permeability based filtering

To filter the molecules obtained from pharmacophore screening on the basis of mycobacterial cell wall permeability, an online server, MycPermCheck^34^ was employed (http://www.mycpermcheck.aksotriffer.pharmazie.uni-wuerzburg.de). Molecules above the cutoff of 0.82 were shortlisted which have a false positive rate of less than 10% as prescribed by the server.

#### 1(d): Docking Studies by using Autodock4.2

The molecules filtered with MycPermCheck were docked at the active site by employing Autodock 4.2^25^. The docking calculations for Autodock 4.2^25^ were carried out on a Fujitsu RX300 S7 Server as master node and Fujitsu CX250 S1 servers as compute nodes (35 no. with CENT-OS version 6).

#### 1(e): Structure similarity based filtering

Structural similarity based filtering was further employed to remove structurally similar molecules. Openbabel software was employed and the molecules above tanimoto coefficient of 0.8 cut-off were retained.

After subjecting the molecules to above-mentioned filters, 22 compounds were purchased from Ambinter c/o Greenpharma, New Orleans, France for subsequent inhibition studies.

#### Strategy 2: Structure similarity based search

The .sdf files of most potent molecules were used as query structures to find structurally similar analogs against the complete NCI library by using the software Openbabel. The tanimoto coefficient with cut-off 0.7 was set and the structurally similar molecules were procured for subsequent *in vitro* studies.

### Resazurin microtiter assay (REMA) based whole cell assay for MIC_90_ determination

MIC_90_ determination was carried out against *M. tuberculosis* cells grown in MB7H9 media (Difco, Middlebrook from Becton Dickinson and Company, Sparks, MD, USA). Appropriate dilutions of the compounds were employed at a concentration range of 1.25 µg/ml–100 µg/ml and incubated with *M. tuberculosis* cells at a final A_600nm_ of 0.02 in MB7H9 in an assay volume of 200 µl and MIC_90_ values were calculated by using REMA^40, 41^ as described previously^42^. Resazurin based assay gives quantitative measurement of the cell viability. Resazurin, a non fluorescent molecule which is blue in colour is reduced to fluorescent resorufin (pink in colour) in the presence of viable cells. The conversion of blue to pink is correlated with viability of the cells. The MIC_90_ is defined as the inhibitor concentration at which fluorescence intensity is decreased by 90% when compared with the appropriate controls. The values were calculated from three independent experiments and represented as Mean ± S.E.

### Cytotoxicity Assays

The cytotoxicity of the compounds was determined by employing REMA using various mammalian cell lines such as THP-1 (human monocytic macrophage cell line), HEK (Human Embryonic Kidney cell line), MDCK (Madine darby canine kidney cell line) and HepG2 (Human hepatocellular carcinoma cell line). 1 × 10^4^ cells/well were employed in a sterilized 96-well plate containing serial dilutions of compounds ranging from 1 µg/ml to 200 µg/ml concentration, in their respective media (RPMI for THP-1, DMEM for HEK and HepG2 and MEM for MDCK) with 10% fetal bovine serum and Antibiotic-Antimycotic mix (Invitrogen Corporation, Carlsbad, California, USA). IC_50_ is defined as the concentration of the compound that causes 50% reduction in the fluorescence intensity indicating 50% viable cells. IC_50_ values were determined from the results of at least two independent tests and Mean ± S.E.M was calculated from the inhibition curve using GraphPad Prism 5.

### Evaluation of the compounds to inhibit the intracellular *M. tuberculosis*

To evaluate the inhibitory potential of the compounds against the growth of intracellular bacteria, *M. tuberculosis* H37Rv was grown till an Abs_600nm_ of 1 to 1.2 and simultaneously, THP-1 cells were grown and activated by the addition of 30 nm phorbol myristic acetate (PMA) followed by incubation at 37 °C for 16 hours in the presence of 5% CO_2_. Media was discarded the next day and the adhered THP-1 cells were washed once with RPMI medium. The cells were scraped, harvested and counted by trypan blue exclusion staining. Simultaneously, *M. tuberculosis* cells were harvested at 4,500 rpm for 10 minutes and washed twice with MB7H9 medium. The *M. tuberculosis* cells were resuspended in RPMI medium and 0.5-mm glass beads were added followed by vortexing for 15 minutes to make a single cell suspension. The suspension was judiciously centrifuged to remove any clumps. Activated THP-1 cells were infected with *M. tuberculosis* in suspension at a multiplicity of infection (MOI) of 5:1 (bacteria/macrophage) at 37 °C with a constant shaking of 100 rpm. Following infection for two hours, the cells were harvested, washed and re suspended again in RPMI medium with addition of Amikacin (200 μg/ml) followed by incubation at 37 °C for 1 hour with constant shaking at 100 rpm to remove any extracellular bacilli. After the Amikacin treatment, cells were harvested, washed and 1 × 10^4^ cells were seeded in each well containing appropriate dilutions of the compounds in a final volume of 250 μl (I-33 and I-34–100 to 500 μg/ml, I-108–25 μg/ml to 150 μg/ml, Rifampicin − 2.5 μg/ml to 10 μg/ml). Simultaneously, 1 × 10^4^ cells of uninfected THP-1 cells were also incubated with the same corresponding dilutions of the compounds to measure the viability of uninfected THP-1 cells in the presence of compounds. The plates were then kept at 37 °C in the presence of 5% CO_2_ for five days and on the 5^th^ day, media was removed from the wells followed by the addition of 100 μl sterile water for 2 hours at 37 °C to lyse the THP-1 cells. The cells were then vigorously pipetted and the entire volume of 100 μl was spotted on MB7H11 agar plates containing oleic acid-albumin-dextrose-catalase (OADC) followed by the incubation at 37 °C for three to four weeks to analyze the inhibition of bacterial growth. Also, 30 μl of 0.01% solution of resazurin was added to uninfected THP-1 plates and the growth measurement was analyzed the next day by alamar blue assay.

## Electronic supplementary material

Supplementary information
