# Supplementary material for: Virtual Screening, pharmacophore development and structure based similarity search to identify inhibitors against IdeR, a transcription factor of Mycobacterium tuberculosis
Source: Sci Rep. 2017 Jul 5;7:4653. doi: 10.1038/s41598-017-04748-9 (PMC5498548; doi:10.1038/s41598-017-04748-9)
Supplement: Supplementary file 1 — Supplementary information [file 41598_2017_4748_MOESM1_ESM.doc]

**Supplementary Information**

**Virtual Screening, pharmacophore development and structure based similarity search to identify inhibitors against IdeR, a transcription factor of *Mycobacterium tuberculosis.***

Akshay Rohilla^1^, Garima Khare^1^* and Anil K. Tyagi^1, 2^*

^1^Department of Biochemistry, University of Delhi South Campus, Benito Juarez road, New Delhi 110021, India

^2^Vice Chancellor, Guru Gobind Singh Indraprastha University, Sector 16-C, Dwarka, New Delhi, India.

*Address correspondence to Prof. Anil Tyagi and Dr. Garima Khare.

Department of Biochemistry, University of Delhi South Campus, Benito Juarez Road, New Delhi- 110021, India. Tel.: 91-11-24115209; Fax: 91-11-24115270;

Email: [aniltyagi@south.du.ac.in](mailto:aniltyagi@south.du.ac.in); [garima1822@yahoo.co.in](mailto:garima1822@yahoo.co.in)

**
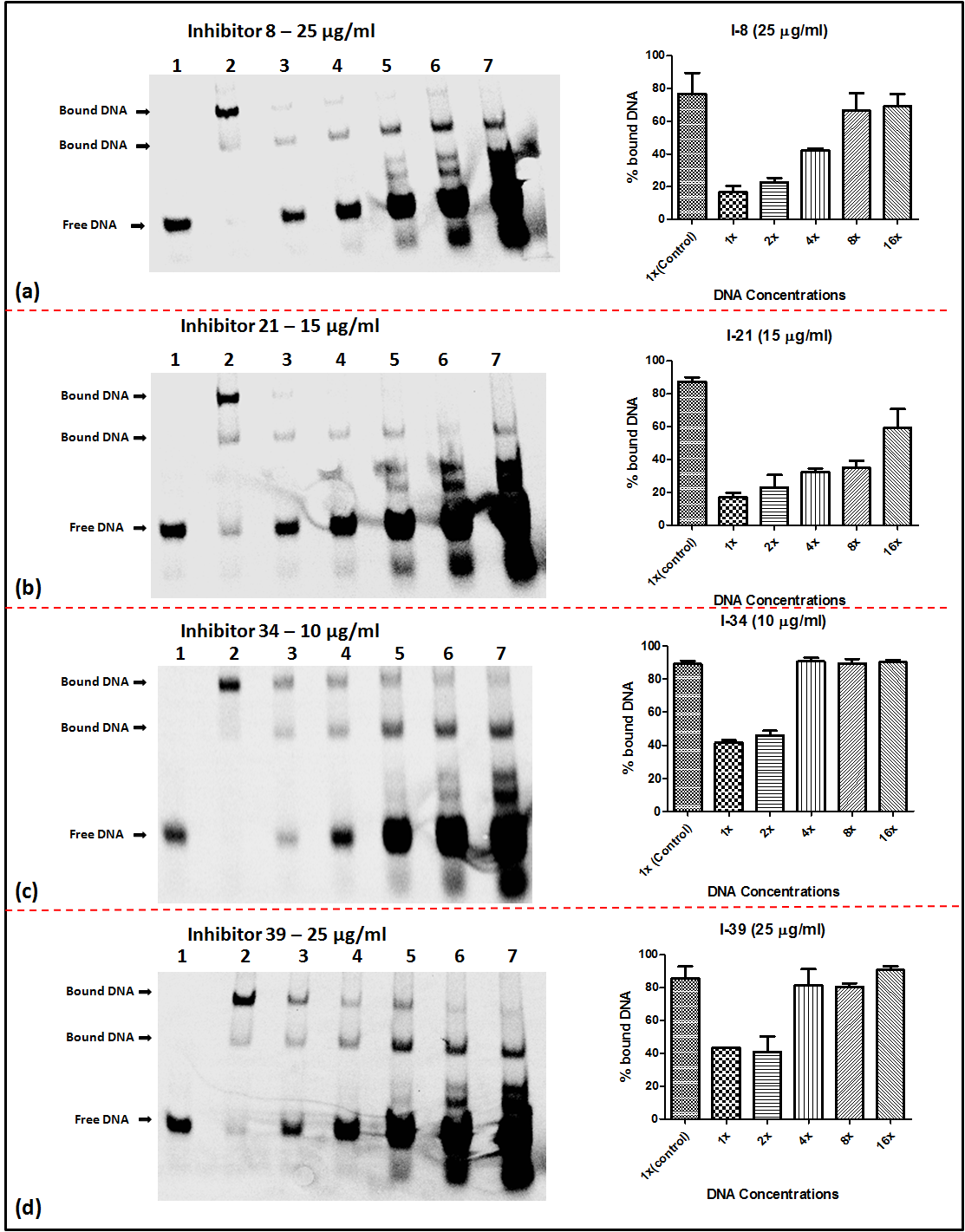
**

**Fig. S1: Competition experiments with compounds I-8, I-21, I-34, I-39 and labeled DNA**. Each gel depicts the alleviation of inhibition exhibited by compound I-8 (a), compound I-21 (b), compound I-34 (c) and compound I- 39 (d) in the presence of increasing DNA. Lane 1- free DNA (0.8 pmoles), lane 2- IdeR + DNA (0.8 pmoles) + DMSO (vehicle control), lane 3- IdeR + DNA (0.8 pmoles) + inhibitor, lane 4- IdeR + DNA (1.6 pmoles) + inhibitor, lane 5- IdeR + DNA (3.2 pmoles) + inhibitor, lane 6- IdeR + DNA (6.4 pmoles) + inhibitor, lane 7- IdeR + DNA (12.8 pmoles) + inhibitor. The bar diagram represents the percent bound DNA at varying DNA concentrations (x represents 0.8 pmoles of labeled DNA). Different inhibitor concentrations were taken for different inhibitors due to their differing IC_50_ values.


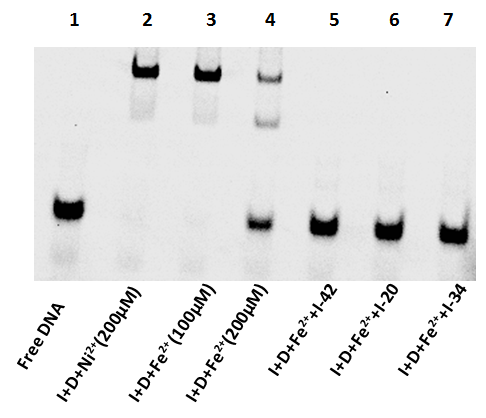


**Fig S2.** **Effect of iron on the DNA binding activity of IdeR and inhibition of the compounds I-42, I-20 and I-34.** This figure depicts the effect of iron on IdeR DNA binding activity as well as inhibition of compounds I-42, I-20 and I-34. I depicts IdeR (0.35 µg), D depicts DNA (0.8 pmoles). Lane 2 denotes Ni^2+^ as a control, lanes 3 and 4 depicts reaction with 100 µM and 200 µM Fe^2+^, respectively, lanes 5, 6 and 7 depicts reaction in the presence of 100 µM Fe^2+^ reaction with 100 µg/ml of compounds I-42, I20 and I-34, respectively.


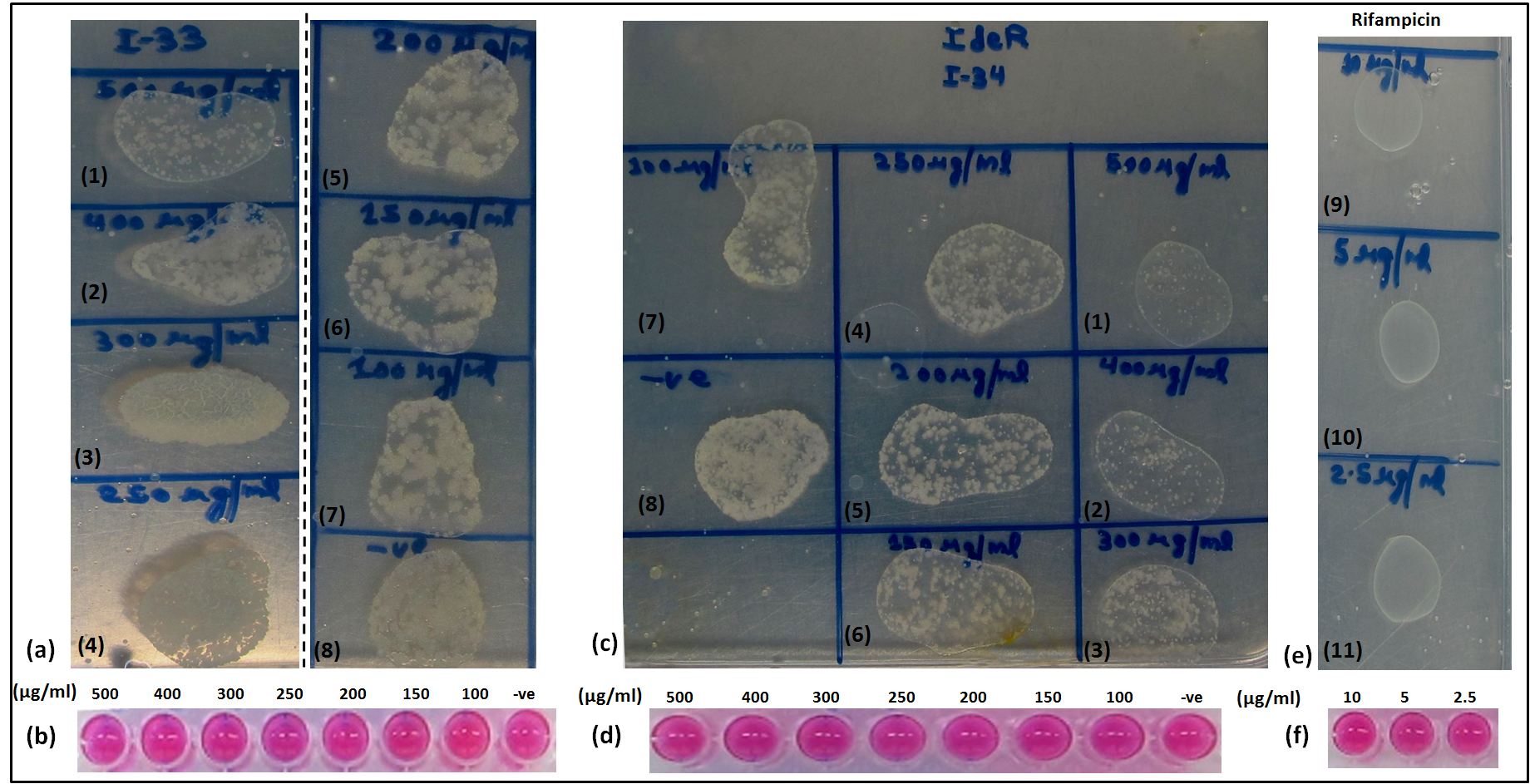


**Fig S3.** **Evaluation of inhibitory potential of compounds I-33 and I-34 against the growth of intraphagosomal *M.tuberculosis***. (a) The influence of varying concentrations of I-33 on the growth of intracellular *M.tuberculosis* as analyzed on the agar plates. (b) Alamar blue assay to measure the viability of uninfected THP-1 cells in the presence of varying concentrations of I-33. (c) The influence of varying concentrations of I-34 on the growth of intracellular *M.tuberculosis*. (d) Alamar blue assay for the measurement of the viability of uninfected THP-1 cells in the presence of varying concentrations of I-34. (e) Analysis of inhibition of growth of intracellular bacteria with varying concentrations of Rifampicin. (f) Alamar blue assay for the measurement of viability of the THP-1 cells in the presence of varying concentration of Rifampicin (The upper lane of panels b, d and f depicts the various concentrations of I-33, I-34 and Rifampicin respectively, which were employed for the alamar blue assay (in μg/ml)). Lanes 1-8 in panel a and c depicts the following concentrations of I-33 and I-34 respectively: Lanes 1- 500 μg/ml, Lanes 2- 400 μg/ml, Lanes 3- 300 μg/ml, Lanes 4- 250 μg/ml, Lanes 5- 200 μg/ml, Lanes 6- 150 μg/ml , Lanes 7- 100 μg/ml , Lanes 8- -ve (only cells), Lane 9- 10 μg/ml rifampicin, Lane 10- 5 μg/ml rifampicin, Lane 11- 2.5 μg/ml rifampicin. The dotted line represents two separate agar plates.

**Table S1. Details of inhibitors resulting from the initial screening of the filtered NCI library.** (I prefix is given to the compounds obtained from the initial docking studies carried out by Autodock 4.2).

| IdeR  Inhibitors | NSC ID | Autodock Score (kcal/mol) | IC_50_  (µg/ml) | Structure | M.Wt | IC_50_  (µM) |
| --- | --- | --- | --- | --- | --- | --- |
| I-8 | 65748 | -6.4 | 23.9±1.69 | 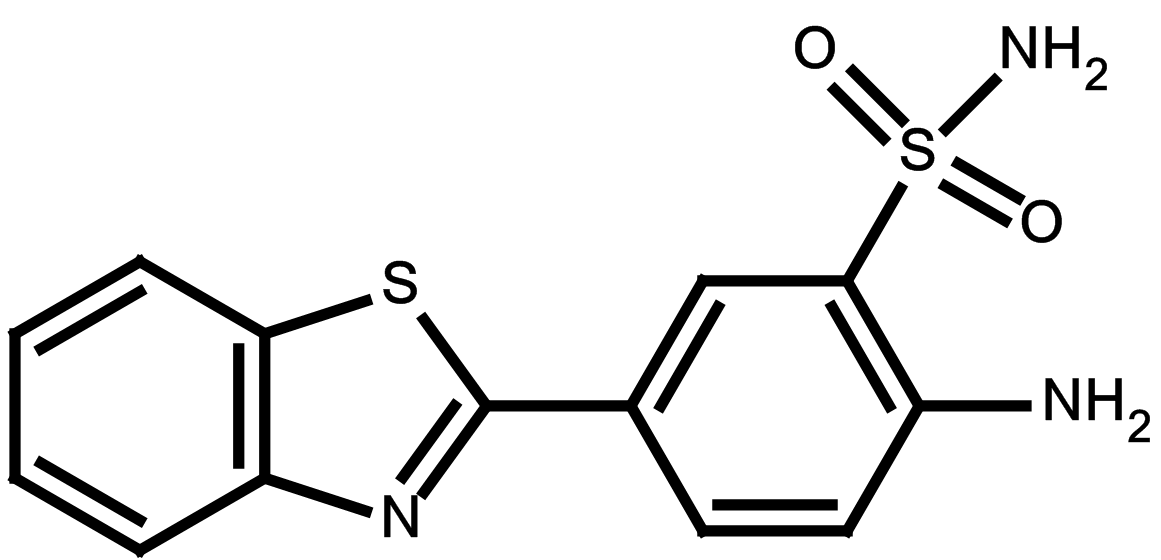**2amino-5-(1,3-benzothiazol-2-yl) benzene sulfonic acid**  **ammoniate** | 323 | 74 |
| I-18 | 201773 | -6.5 | 14±1.52 | 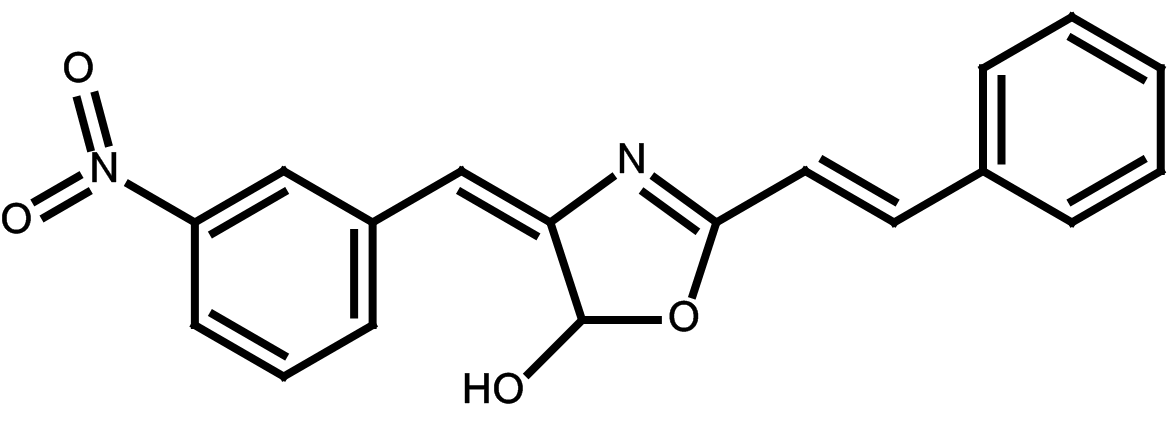  **4-(3-nitrobenzylidene)-2-[2-phenylethenyl]-1,3-oxazol5(4H)-one** | 320 | 43.75 |
| I-20 | 281033 | -6.4 | 2.44±1.41 | 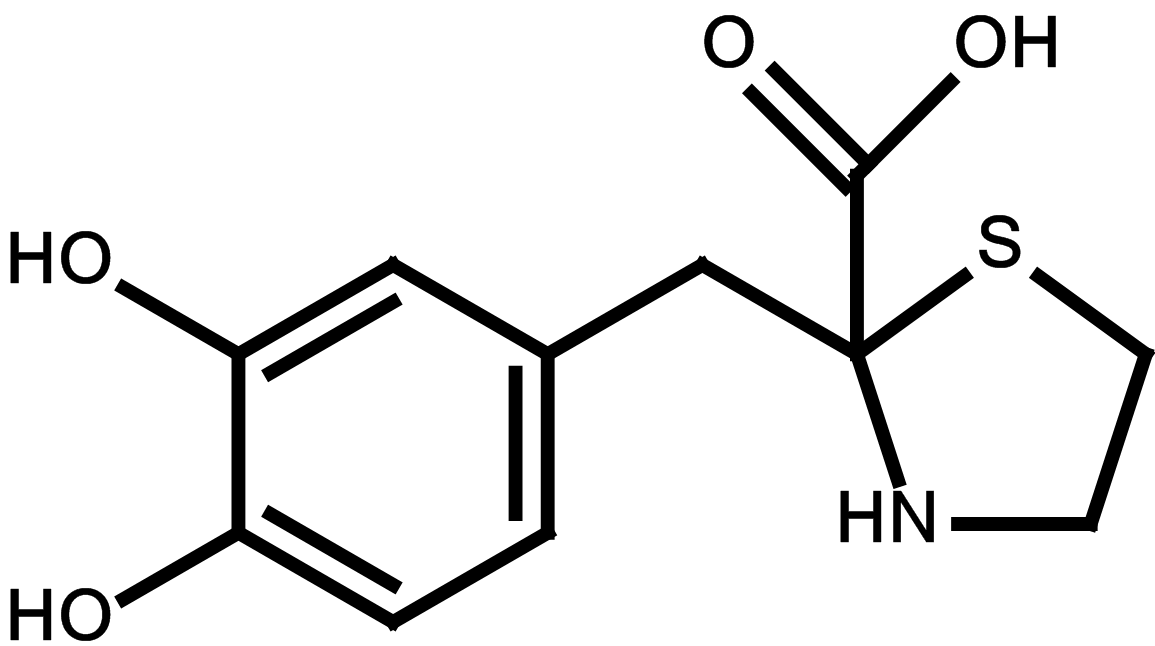  **2-(3,4-dihydroxybenzyl)-1,3-thiazolidine-2-carboxylic acid** | 255 | 9.56 |
| I-21 | 303600 | -6.5 | 5.48±2.01 | 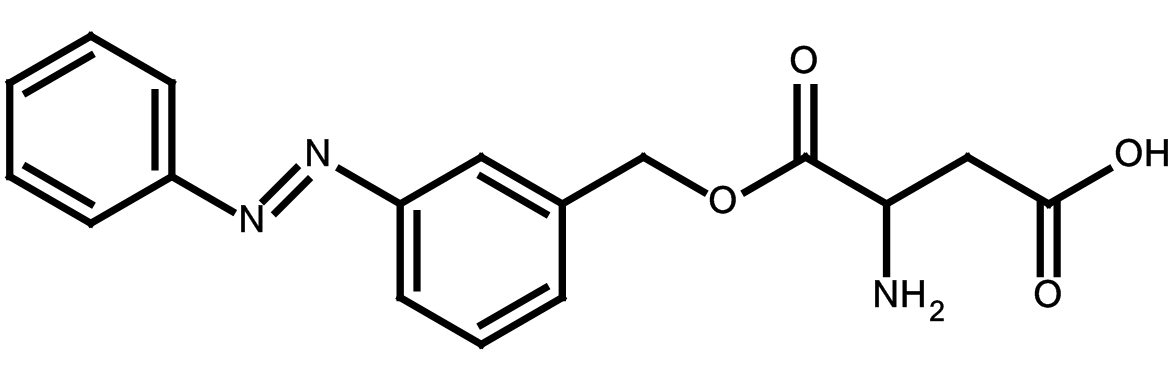**3-amino-4-oxo-4-({3-[phenyldiazenyl]benzyl}oxybutanoic acid** | 327 | 16.75 |
| I-33 | 662443 | -6.5 | 21.72±1.76 | 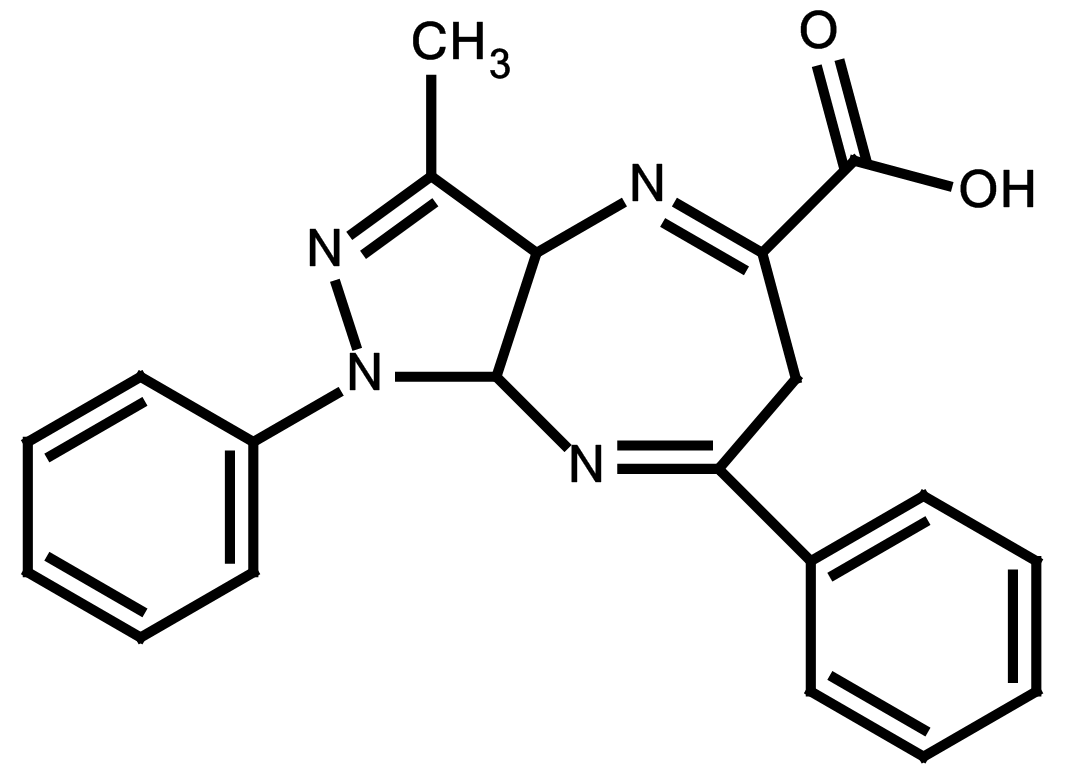  **3-methyl-1,7-diphenyl-1,6-dihydropyrazolo[3,4-b][1,4]diazepine-5-carboxylic acid** | 344 | 63 |
| I-34 | 662444 | -6.59 | 6.1±1.79 | 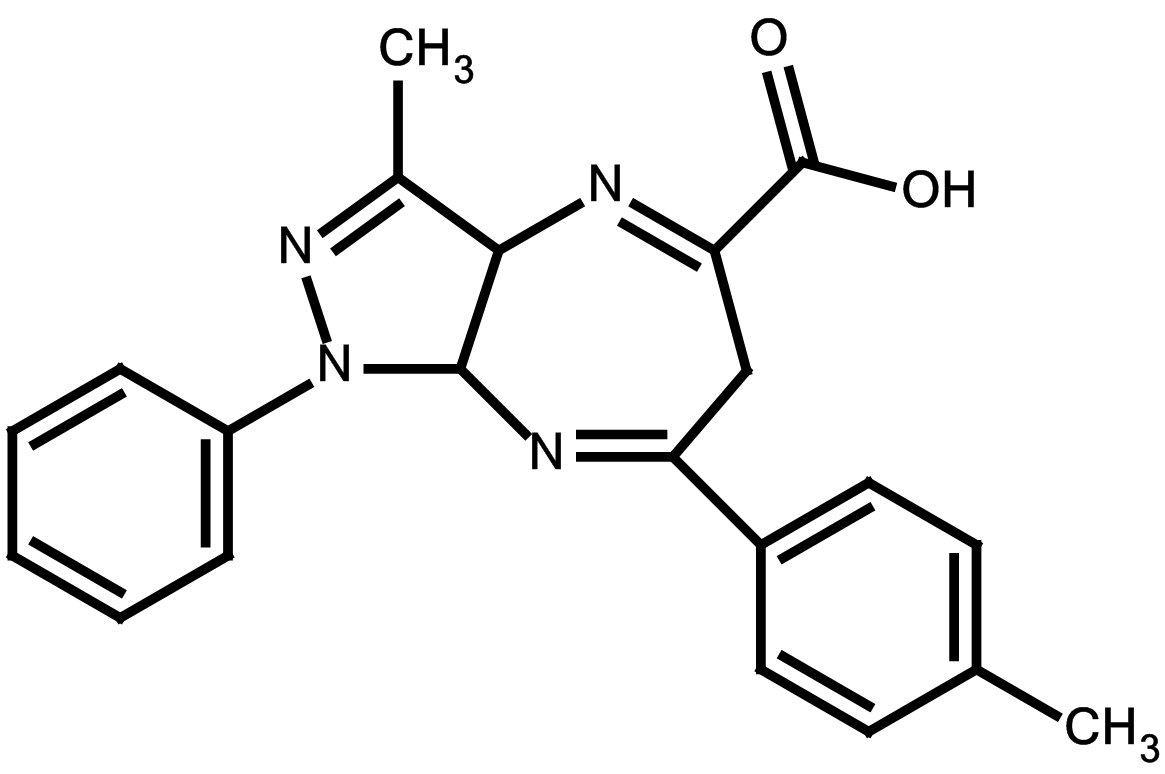  **3-methyl-7-(4-methylphenyl)-1-phenyl-1,6-dihydropyrazolo[3,4-b][1,4]diazepine-5-carboxylic acid** | 358 | 17 |
| I-38 | 673333 | -6.43 | 100±3.176 | 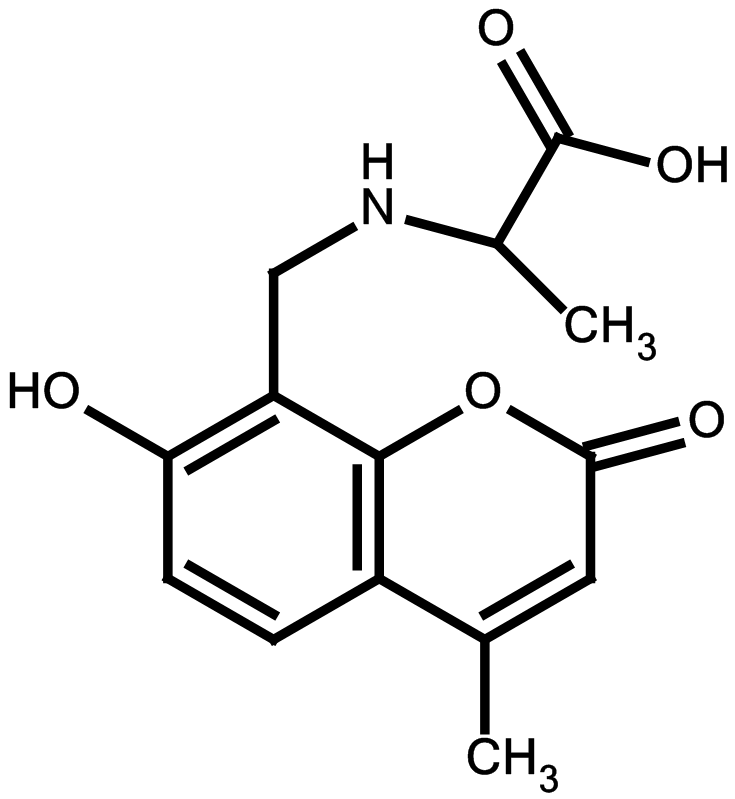  **2-{[6-hydroxy-4-methyl-2-oxo-2H-chromen-8-yl)methyl]amino}propanoic acid** | 277 | 361 |
| I-39 | 673342 | -6.81 | 14.2±1.29 | 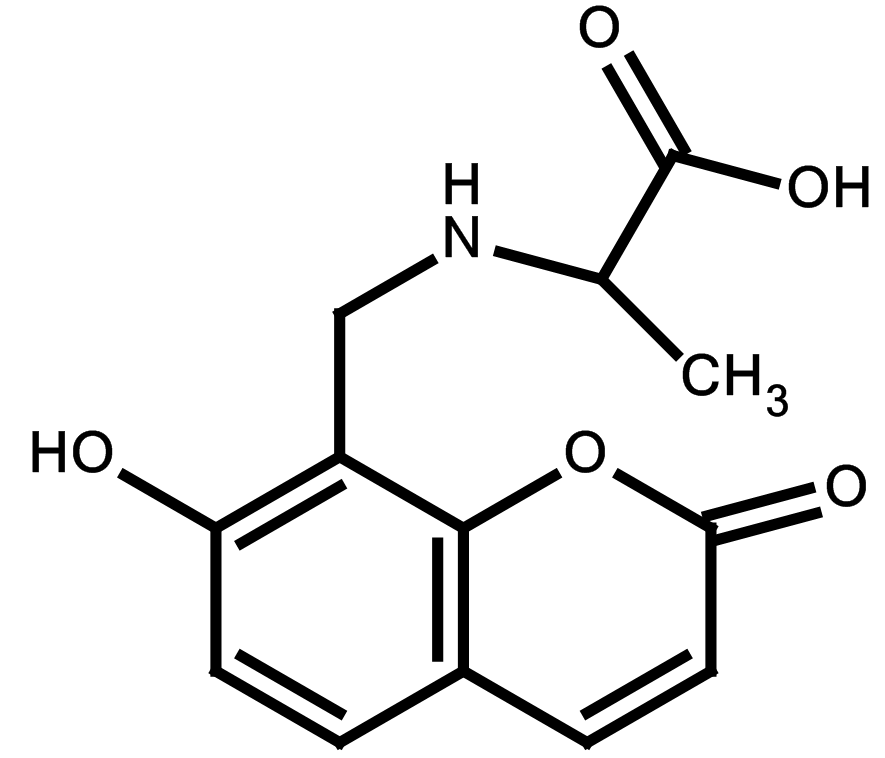  **{[7-hydroxy-2-oxo-2H-chromen-8-yl)methyl]amino}propanoic acid** | 263 | 54 |
| I-85 | 28489 | -6.19 | 28.4±2.43 | 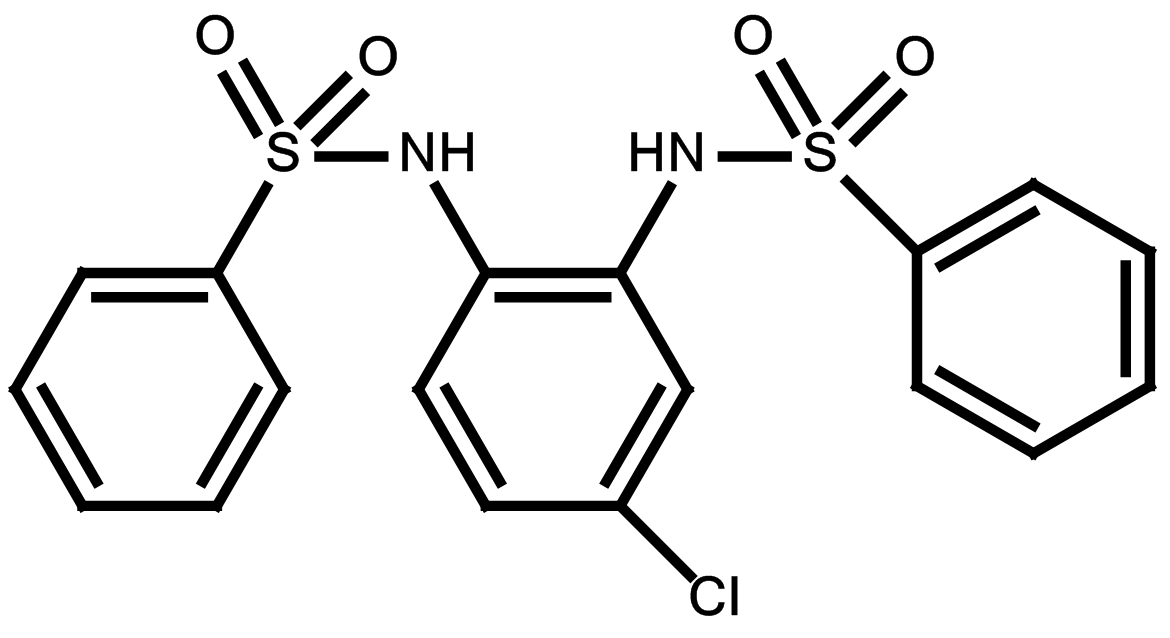  **N,N`-(4-chlorobenzene-1,2-diyl)dibenzenesulfonamide** | 423 | 67.13 |
| I-89 | 44417 | -6.26 | 29.1±3.16 | 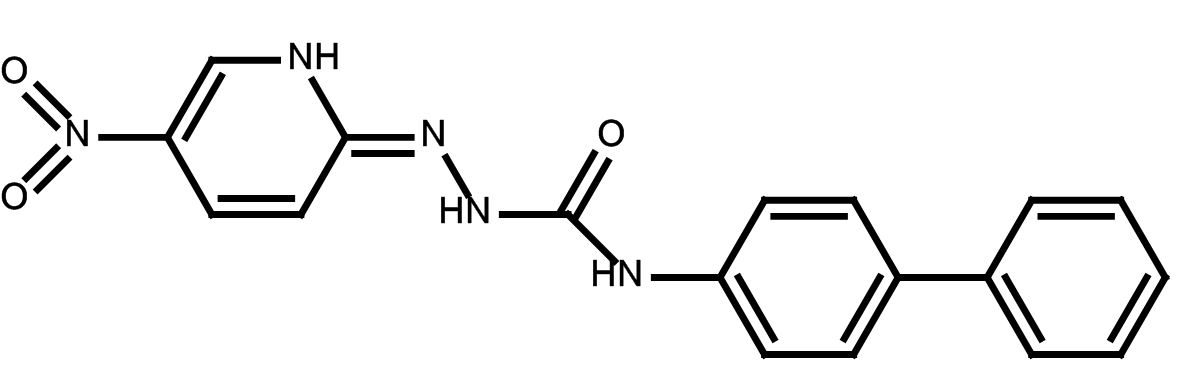  **N-(biphenyl-4-yl)-2-(5-nitropyridin-2(1H)-ylidene)hydrazinecarboxamide** | 349 | 83.38 |
| I-90 | 45558 | -6.35 | 45.27±1.3 | 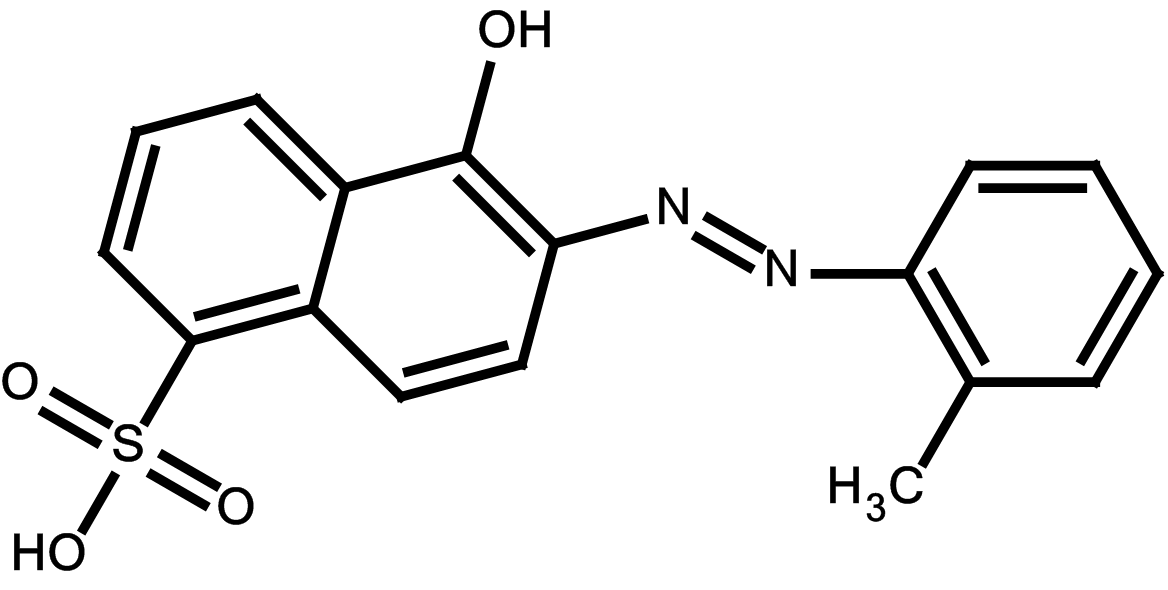  **1-Naphthalenesulfonic acid,5-hydroxy-6-[(2-methylphenyl) azo]-monosodium salt** | 365 | 123.83 |
| I-91 | 46715 | -6.30 | 74.8± 1.8 | 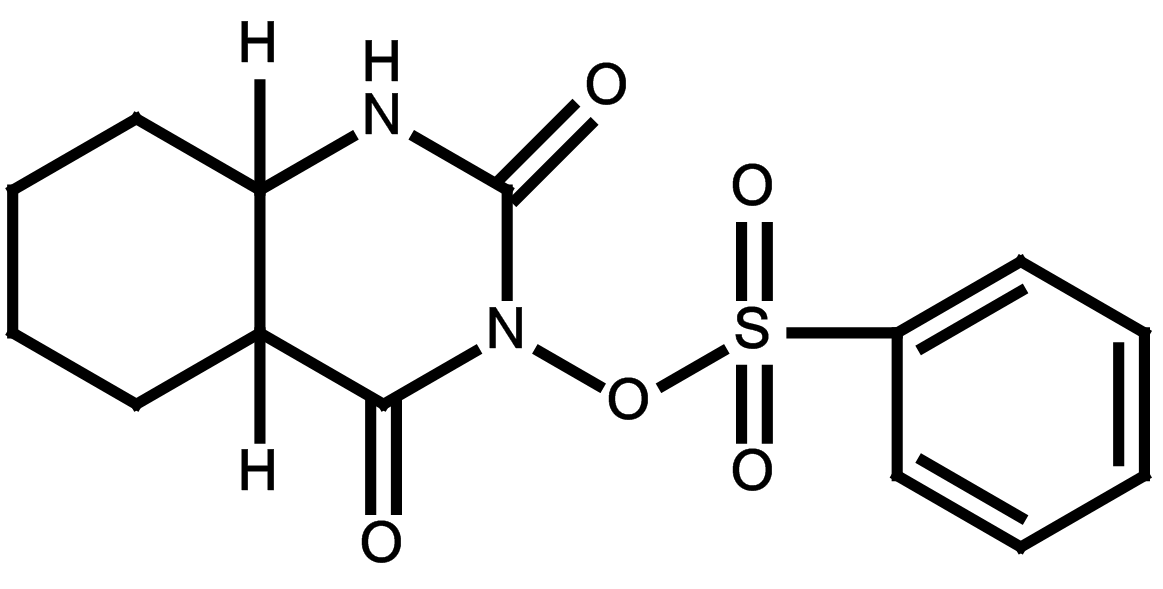  **(4As,8As)-3-[(phenylsulfonyl)oxy]hexahydroquinazoline-2,4(1H,3H)-dione** | 324 | 230.8 |
| I-92 | 56424 | -6.28 | 58.53±0.16 | 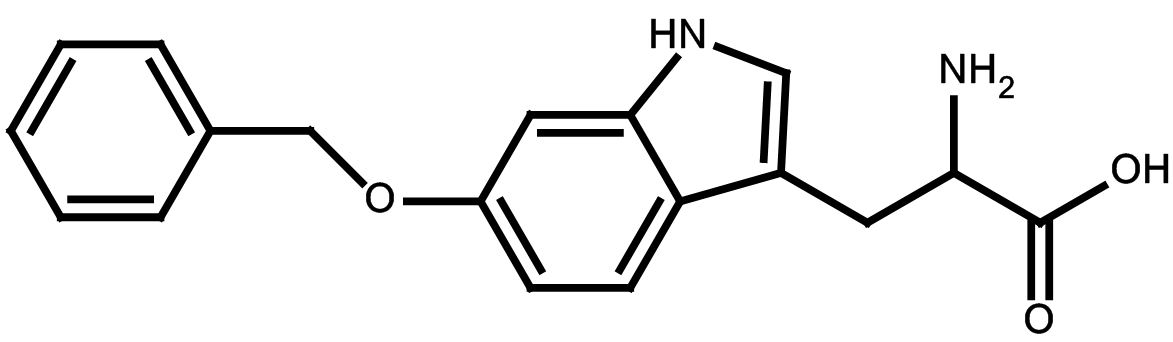  **2-amino-3-[6-(benzyloxy)-1H-indol-3-yl]propanoic acid** | 310 | 188.7 |
| I-93 | 64058 | -6.20 | 54.1±  1.61 | 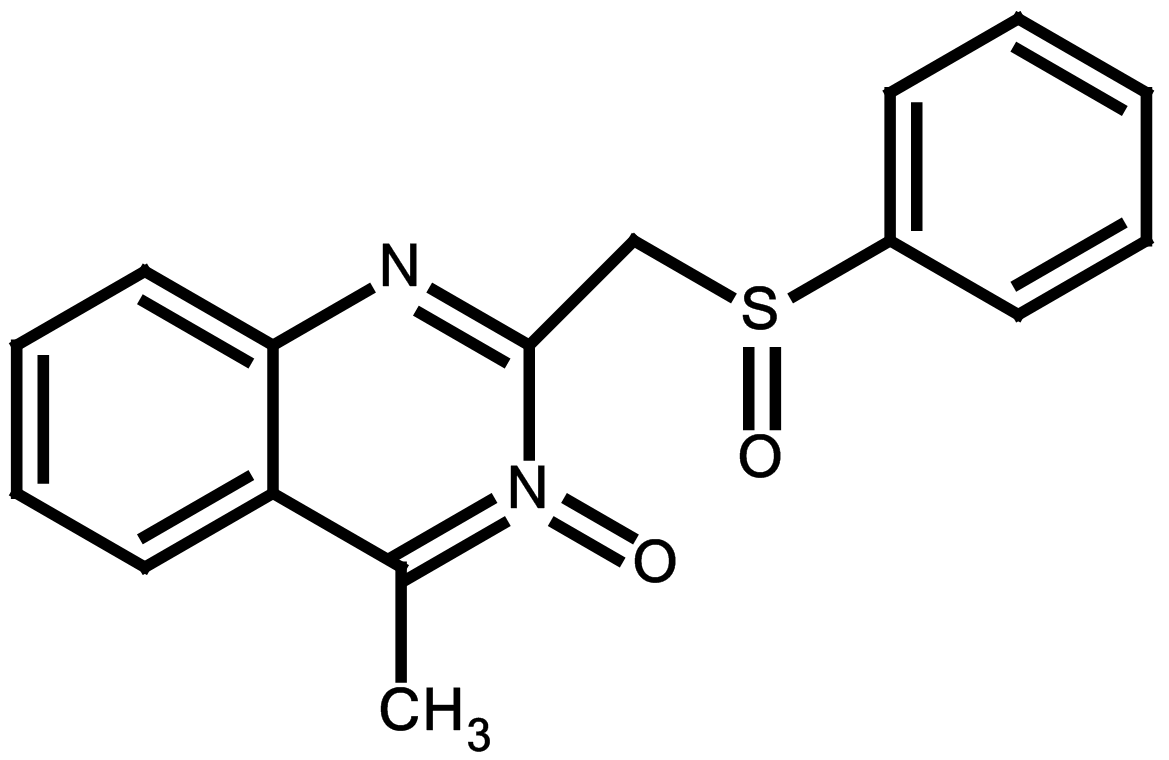  **4-methyl-2-[(phenylsulfinyl)methyl]quinazoline 3-oxide** | 298 | 181.54 |
| I-103 | 153166 | -4.39 | 33.31±2.09 | 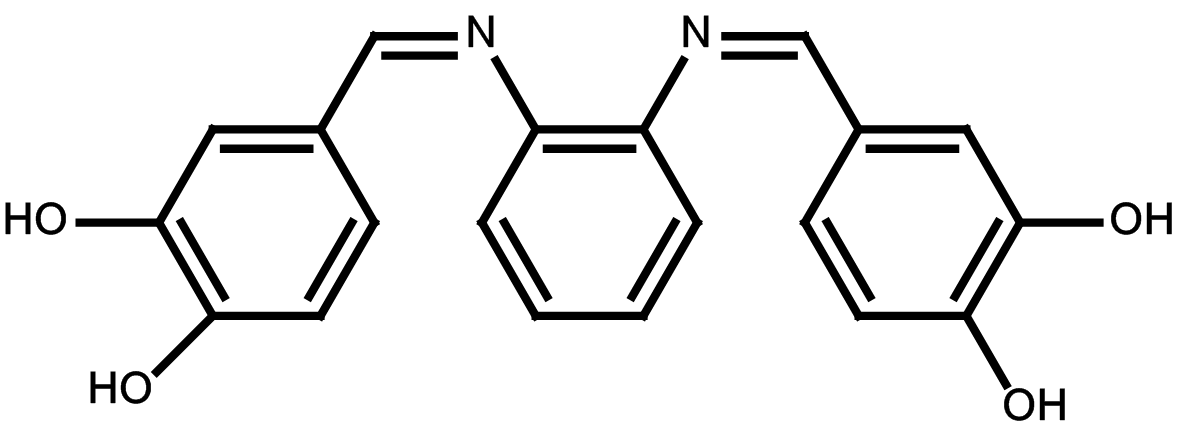  **4,4`-{benzene-1,2-diylbis[nitrilo(Z)methylylidene]}dibenzene-1,2-diol** | 348 | 95.6 |
| I-108 | 282699 | -6.20 | 24.3±2.71 | 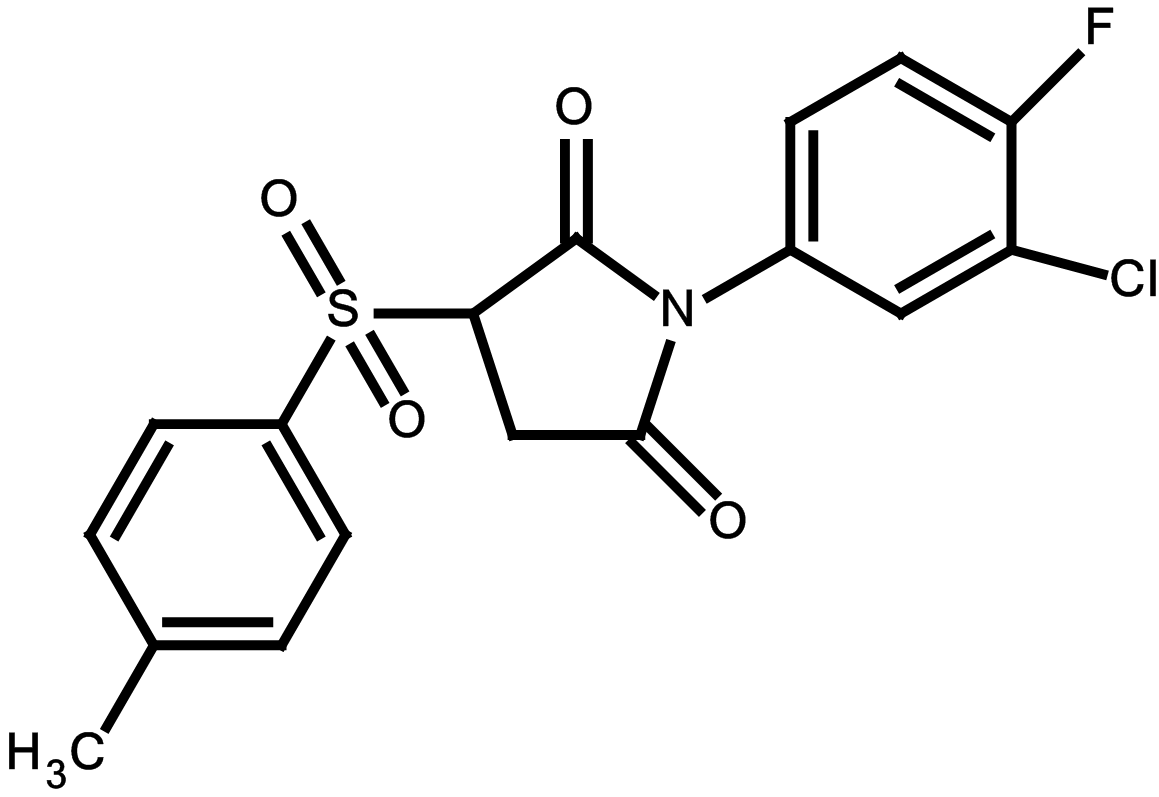**1-(3-chloro-4-fluorophenyl)-3-[(4-methylphenyl)sulfonyl]pyrrolidine-2,5-dione** | 382 | 63.61 |
| I-112 | 360506 | -6.31 | 52.19±  1.6 | 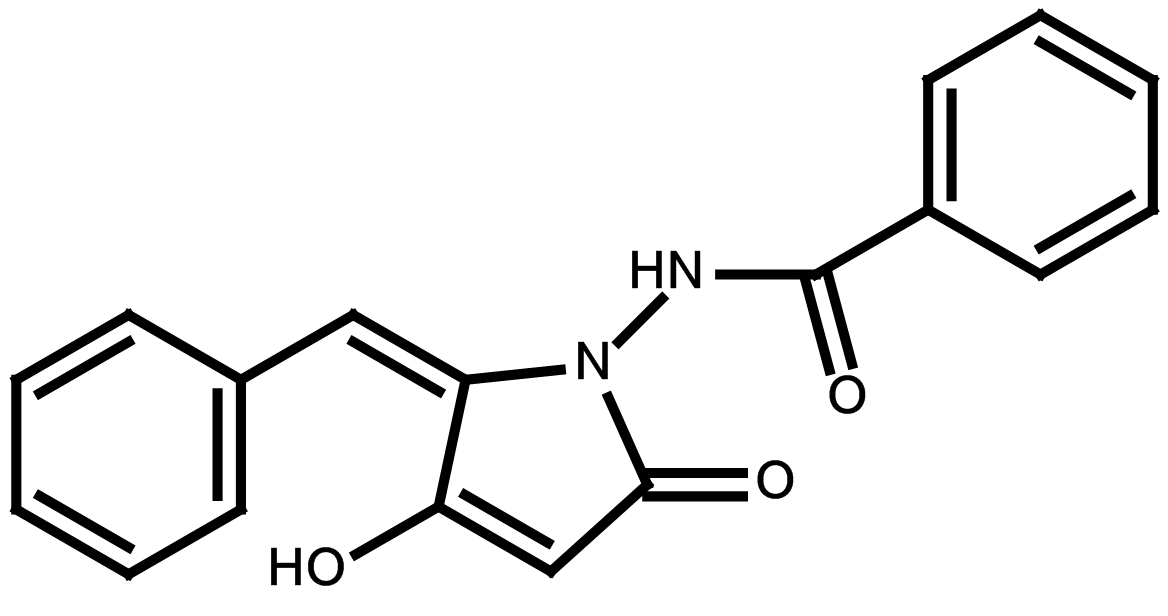  **N-[2-benzylidene-3-hydroxy-5-oxo-2,5-dihydro-1H-pyrrol-1-yl]benzamide** | 306 | 170.26 |
| I-42 | 12453 | -6.31 | 1±1.667 | 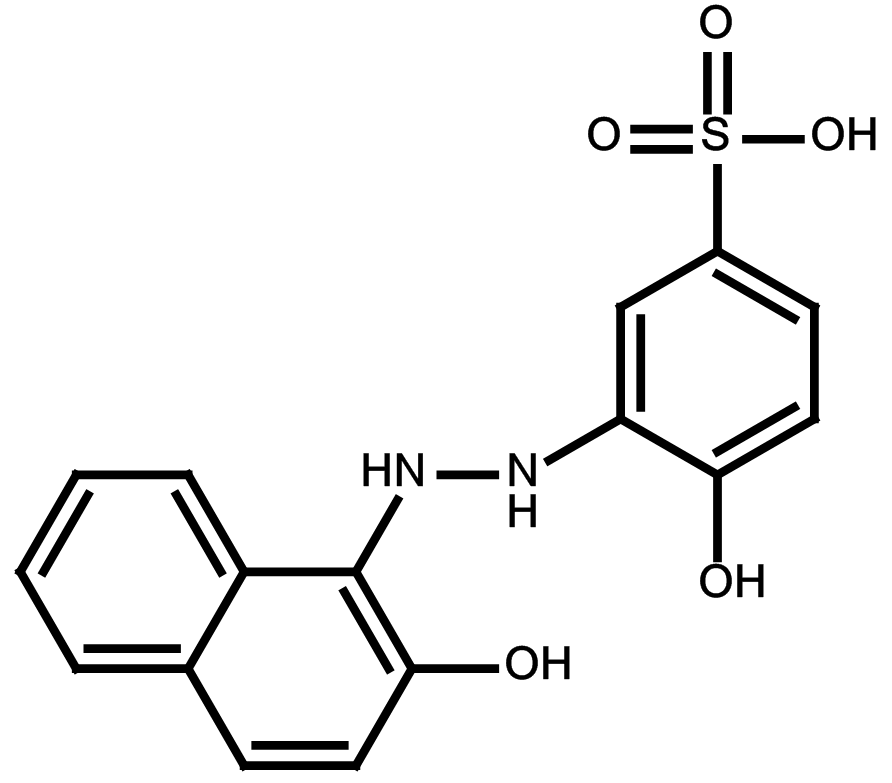**Benzene sulfonic acid,4-hydroxy-3-[(2-hydroxy-1-naphthalenyl)azo]-monosodium salt** | 367 | 2.72 |

**Table S2: List of the molecules exhibiting IC_50_ ≤ 100 µg/ml resulting from structure based similarity search approach. (IS prefix is given to the following compounds obtained from the structure similarity based approach).**

| S.No. | NCI ID | M.Wt | IC_50_ (µg/ml) | Structure | Tanimoto Coefficient | IC_50_ (µM) |
| --- | --- | --- | --- | --- | --- | --- |
| Analogs of I-8 |  |  |  |  |  |  |
| IS-4 | 33755 | 344 | 14.49±2.36 | 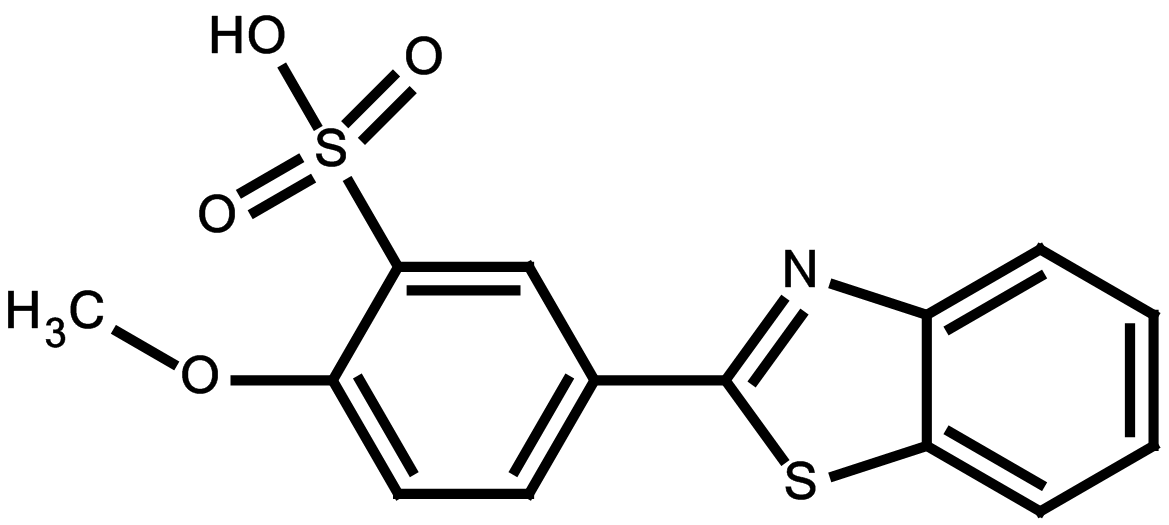  **5-(1,3-benzothiazol-2-yl)-2-methoxybenzenesulfonic acid** | 0.81 | 42.12 |
| IS-32 | 402362 | 323 | 20.94±  2.23 | 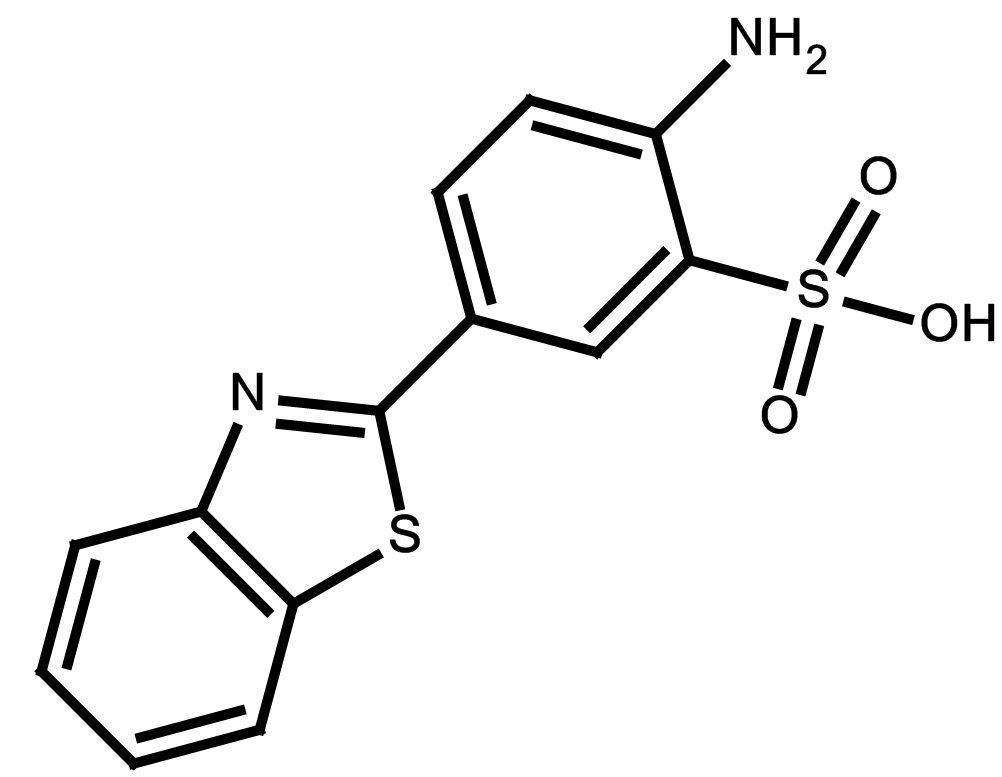  **2-Amino-5-(1,3-benzothiazol-2-yl) benzenesulfonic acid** | 1 | 64.82 |
| IS-54 | 34445 | 226 | 11.2±1.378 | 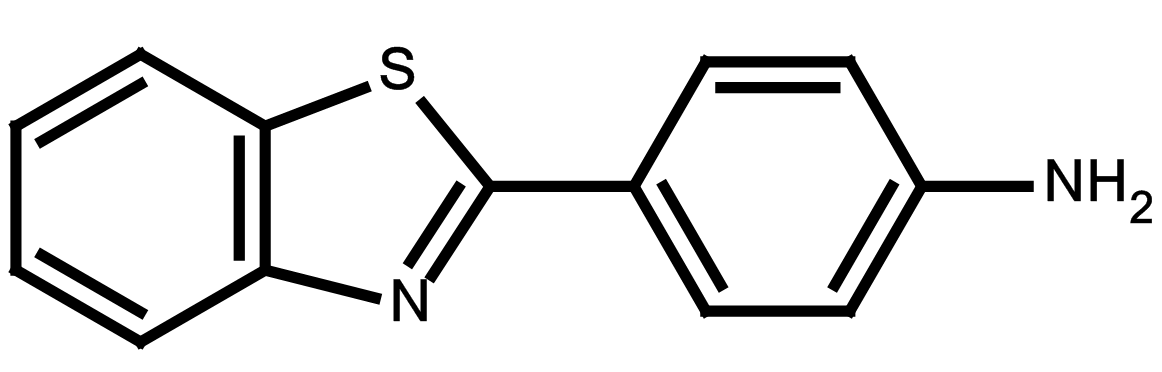  **4-(1,3-benzothiazol-2-yl)aniline** | 0.71 | 49.55 |
| Analogs of I-39 |  |  |  |  |  |  |
| IS-10 | 77040 | 353 | 86.4 ± 3.78 | 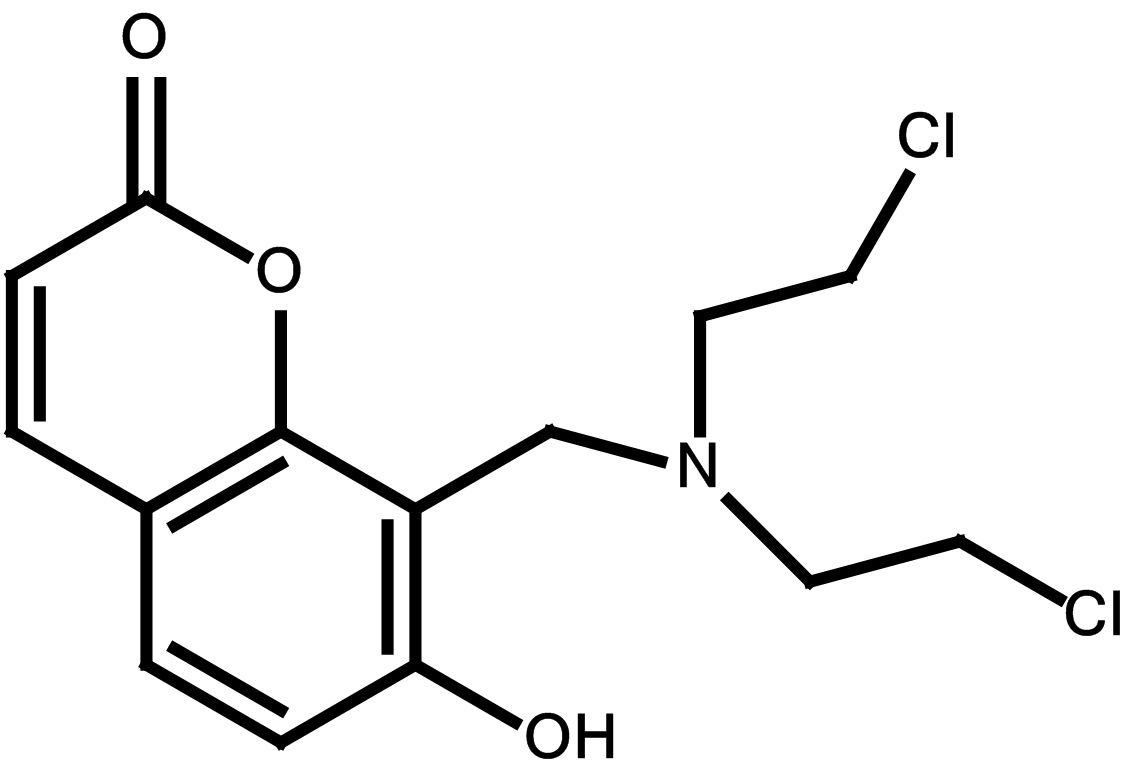**8-{[bis(2-chloroethyl)amino]methyl]}-7-hydroxy-2H-chromen-2-one hydrochloride** | 0.73 | 244.76 |
| IS-40 | 673336 | 301 | 82.7 ± 2.78 | **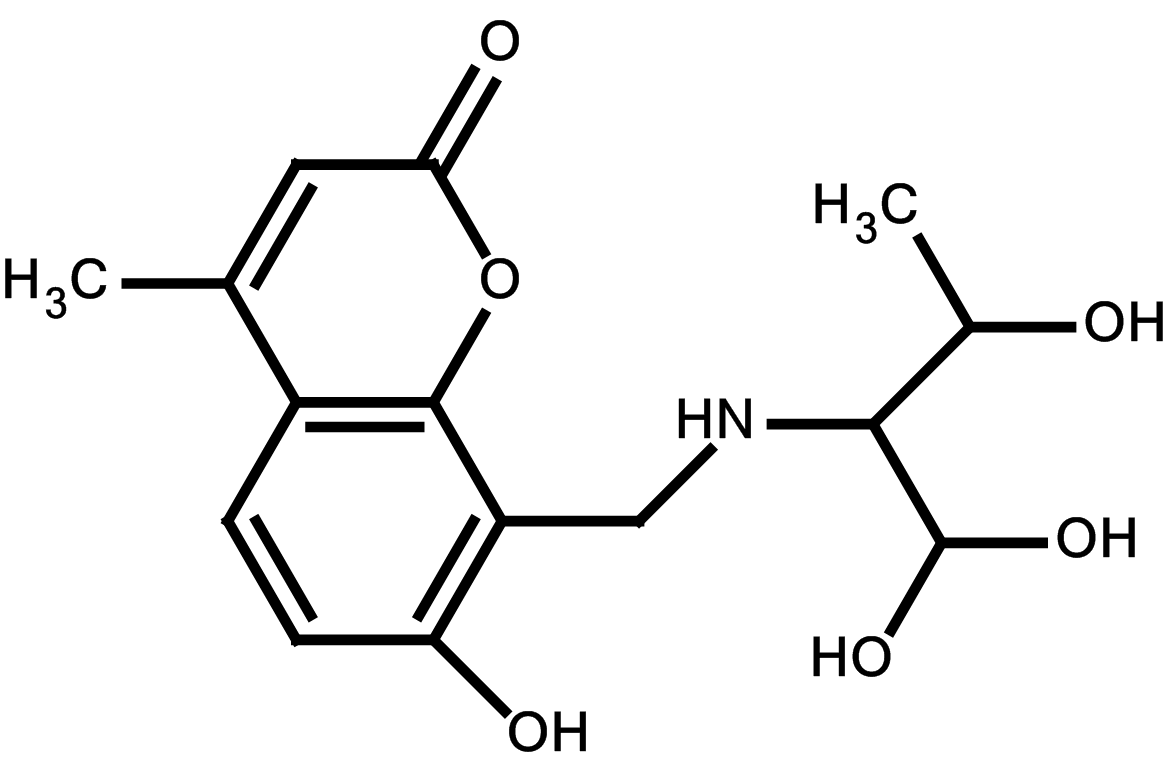**  **3-hydroxy-2-{[(7-hydroxy-4-methyl-2-oxo-2H-chromen-8-yl)methyl]amino}butanoic acid** | 0.8 | 274.75 |
| Analogs of 42 | 12453 |  |  |  |  |  |
| IS-2 | 12448 | 337 | 5.48±1.548 | 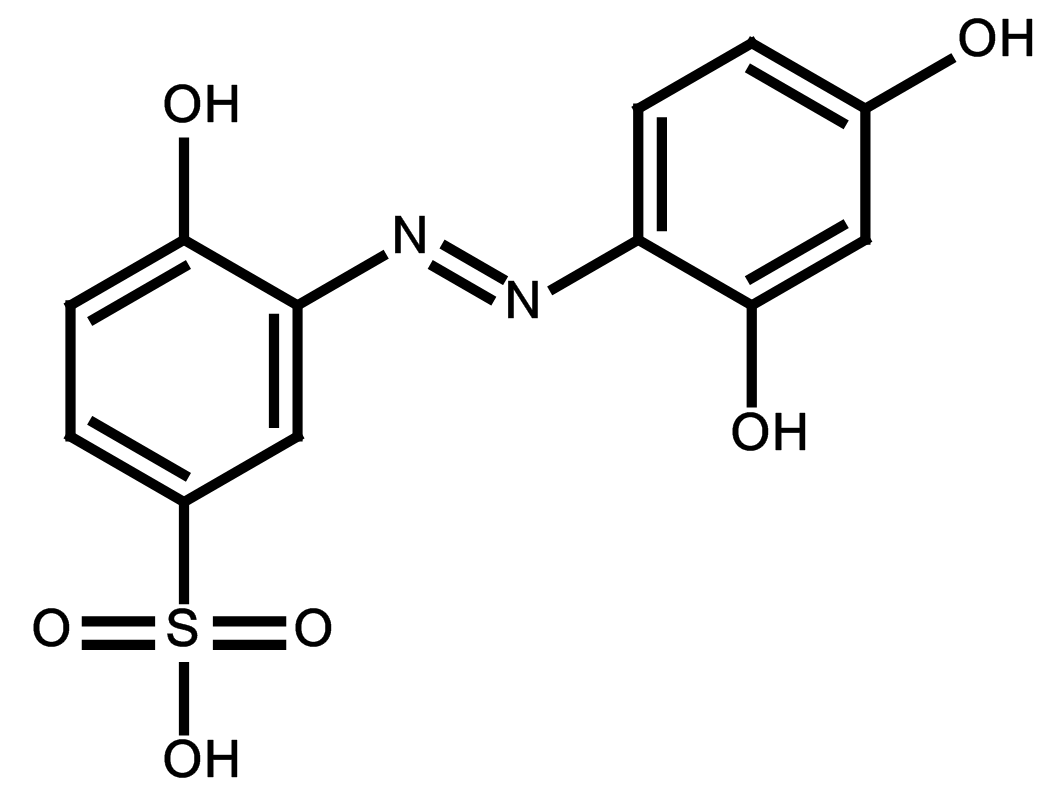  **Benzensulfonic acid, 3-[(2,4- dihydroxyphenyl)azo]-4-hydroxy** | 0.91 | 16.26 |
| IS-6 | 45578 | 401 | 21.66±2.52 | 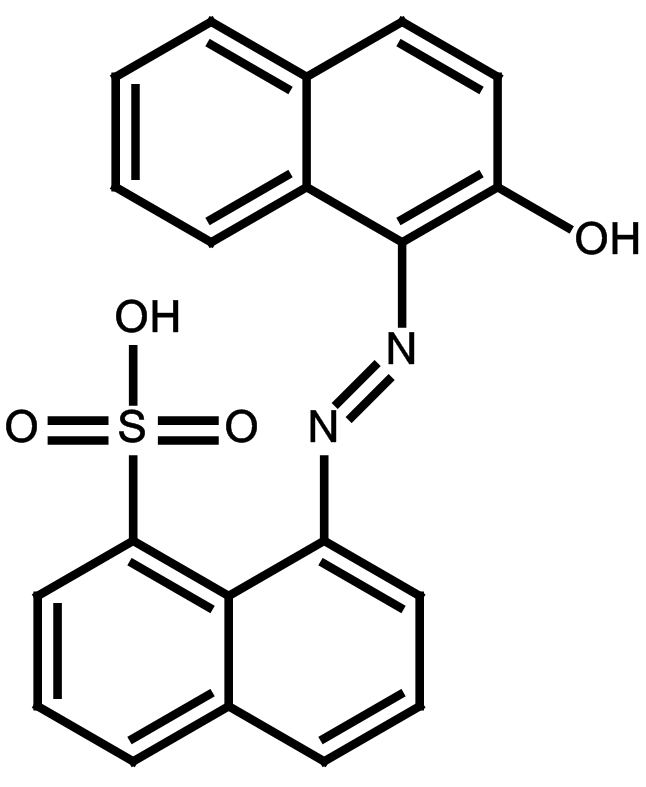  **8-[-(2-hydroxynaphthalen-1-yl)diazenyl]naphthalene-1-sulfonic acid** | 0.92 | 54 |
| IS-7 | 45591 | 559 | 32.13±1.56 | 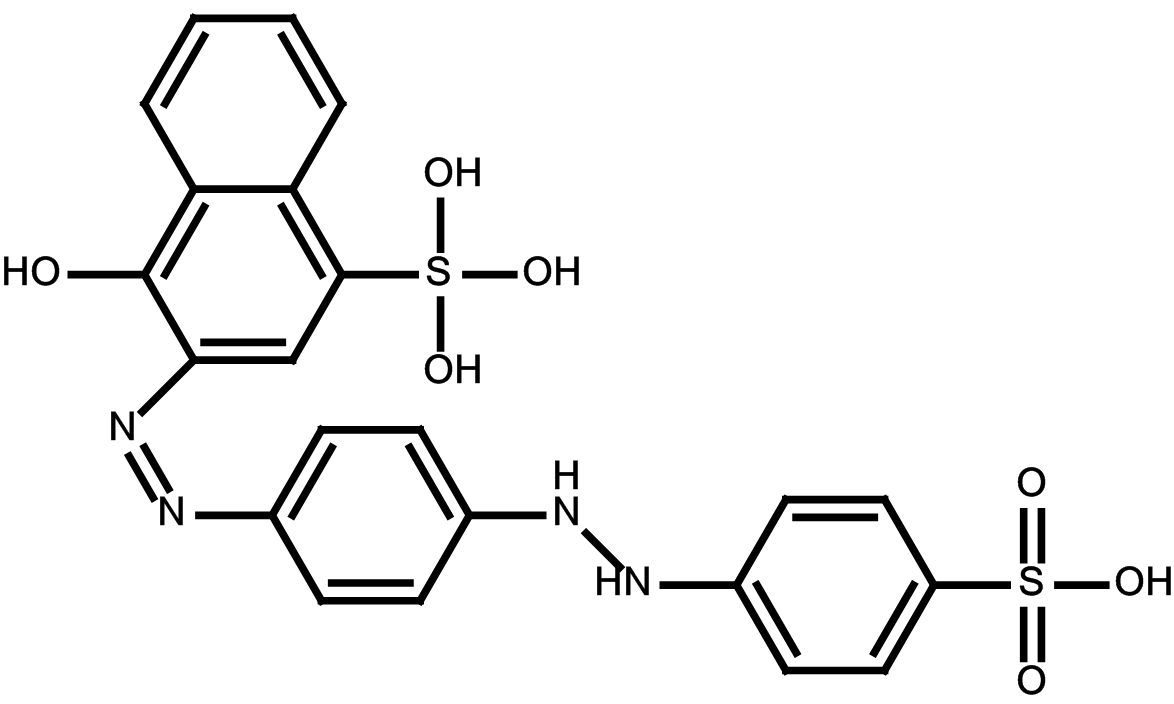  **4-oxo-3-({4-[-(4-sulfophenyl)diazenyl]phenyl}hydrazono)-3,4-dihydro-1-naphtalenesulfonic acid** | 0.9 | 57.47 |
| IS-8 | 47766 | 881 | 0.667±1.82 | 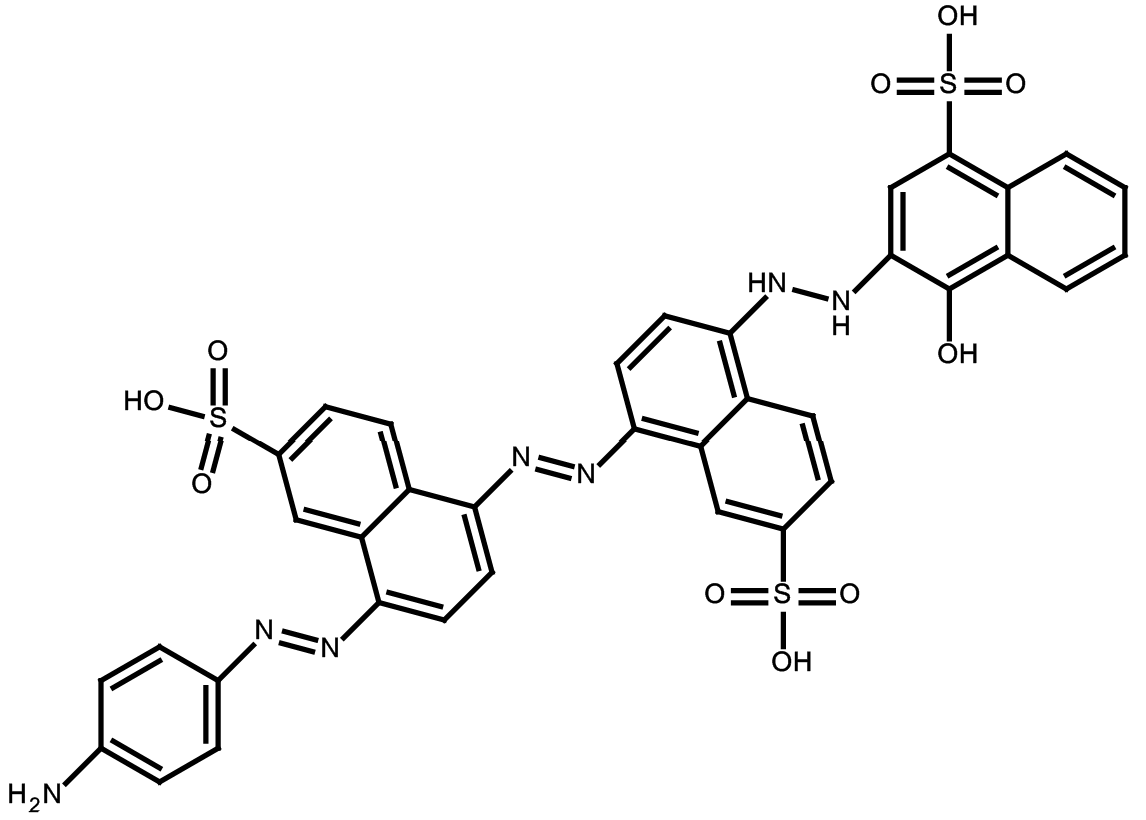**1-Naphthalenesulfonic acid, 3-[[4-[[4-[(4-aminophenyl)azo]-6-sulfo-1-naphthalenyl]azo]-6-sulfo-1-naphthalenyl]azo]-4-hydroxy-,trisodium salt** | 0.9 | 0.75 |
| IS-9 | 75957 | 504 | 13.9±7.37 | 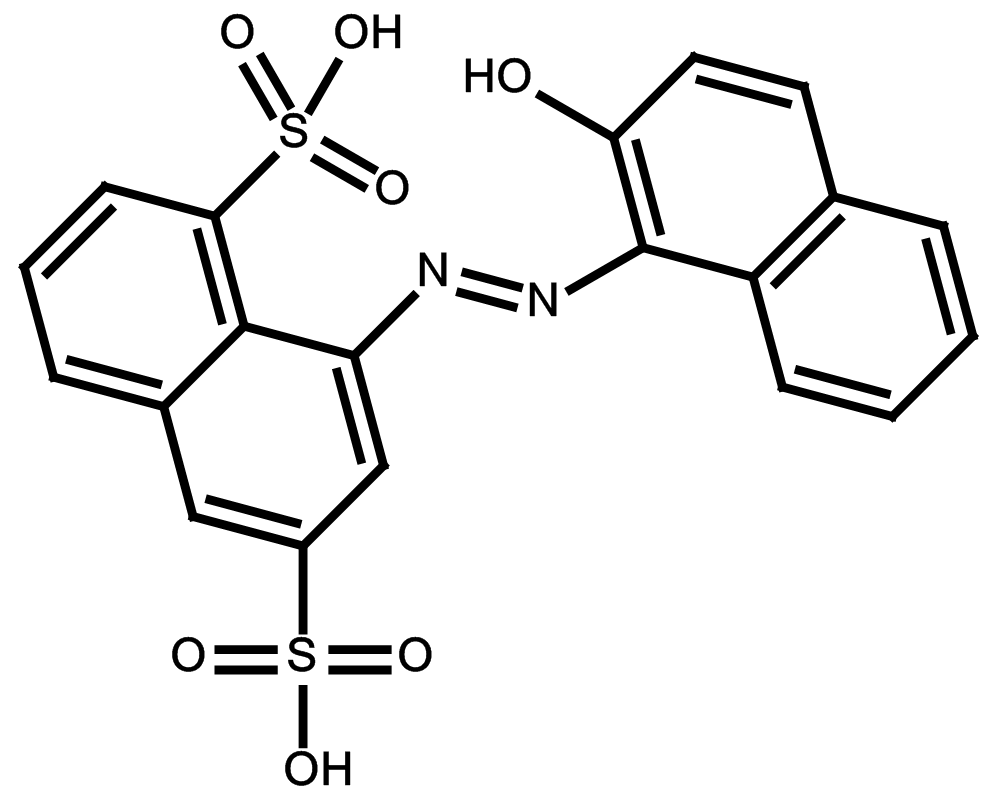**8-[-(2-hydroxynaphthalen-1-yl)diazenyl]naphthalene-1,6-disulfonic acid, disodium salt** | 0.92 | 27.58 |
| IS-12 | 97024 | 310 | 4.69±1.22 | 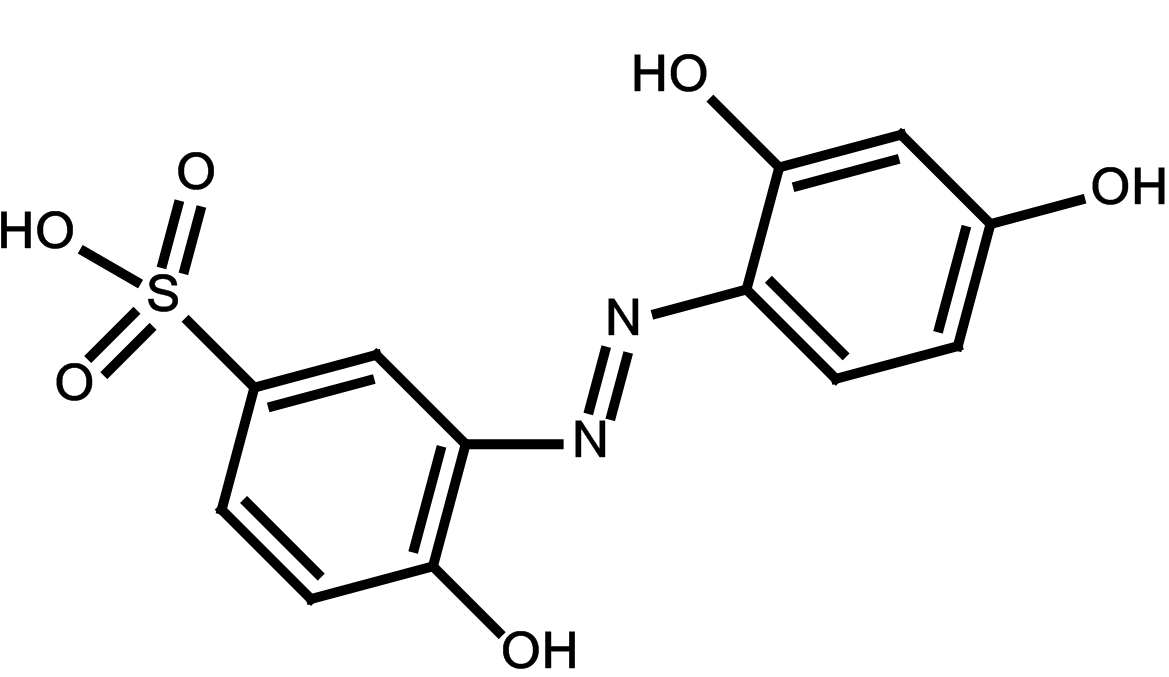  **Benzene sulfonic acid, 3-[(2,4-dihydroxyphenyl)azo]-4-hydroxy** | 0.92 | 15.16 |
| IS-45 | 4299 | 504 | 20.19±  1.74 | 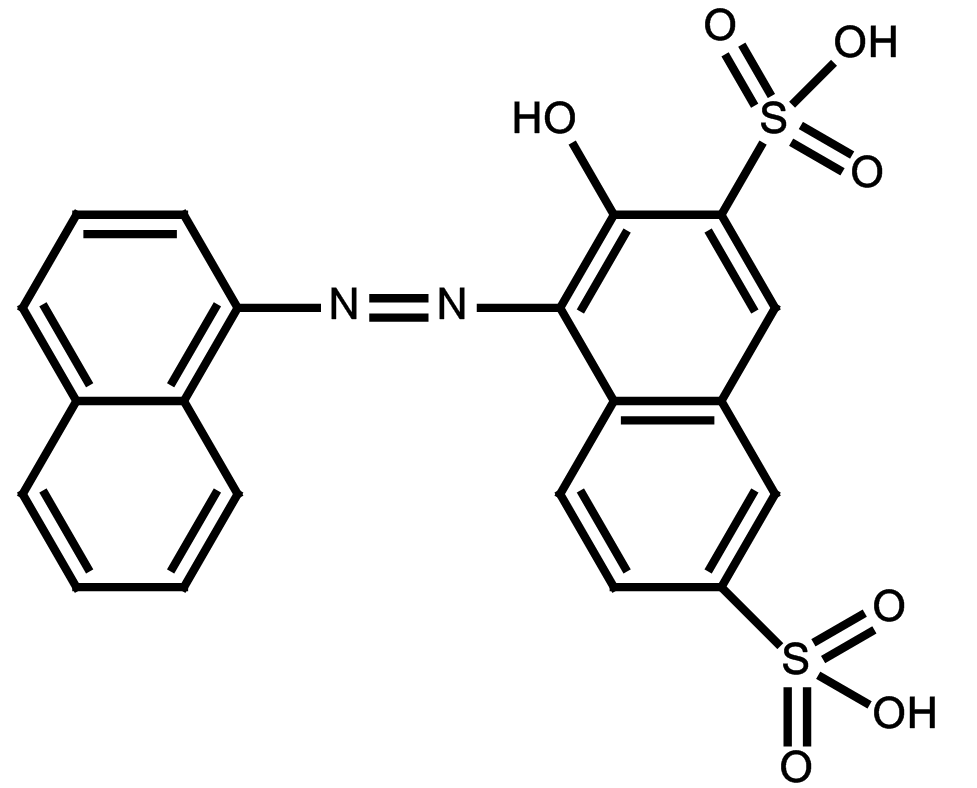  **2,7-naphthalenedisulfonic acid, 3-hydroxy-4-(1-napthalenylazo)** | 0.88 | 40 |
| IS-55 | 45204 | 401 | 6.6±1.95 | 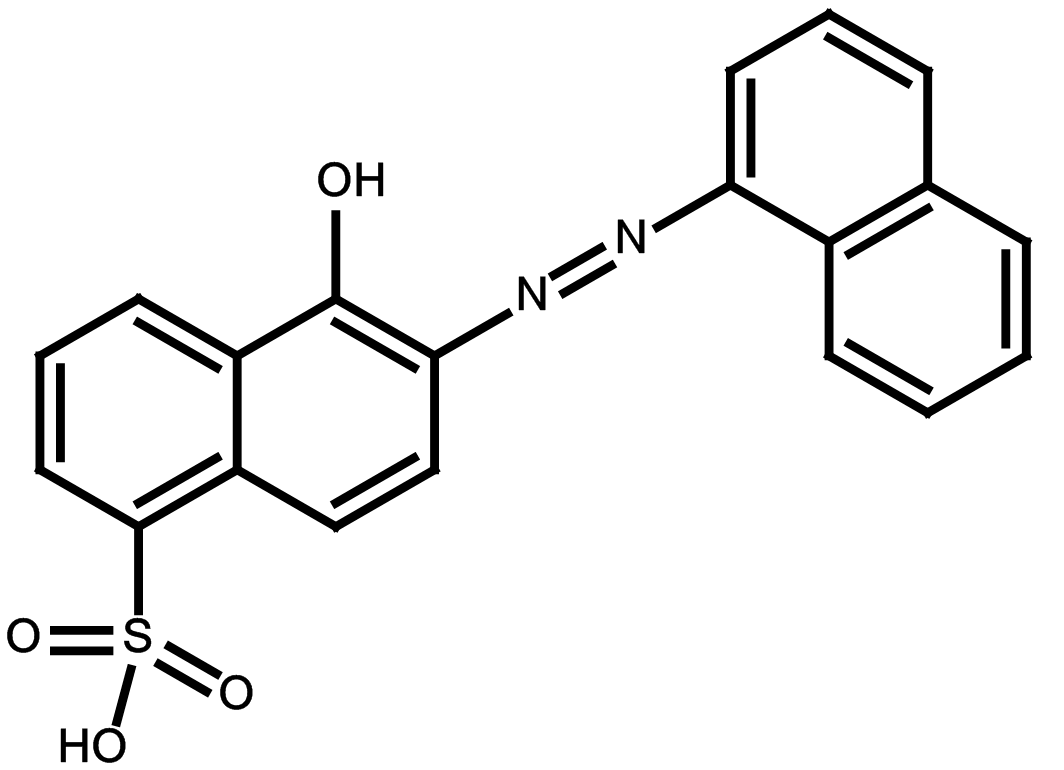  **1-napthalenesulfonic acid,5-hydroxy-6-(1-naphthalenylazo)** | 0.88 | 16.45 |
| IS-59 | 47706 | 381 | 18.64±1.532 | 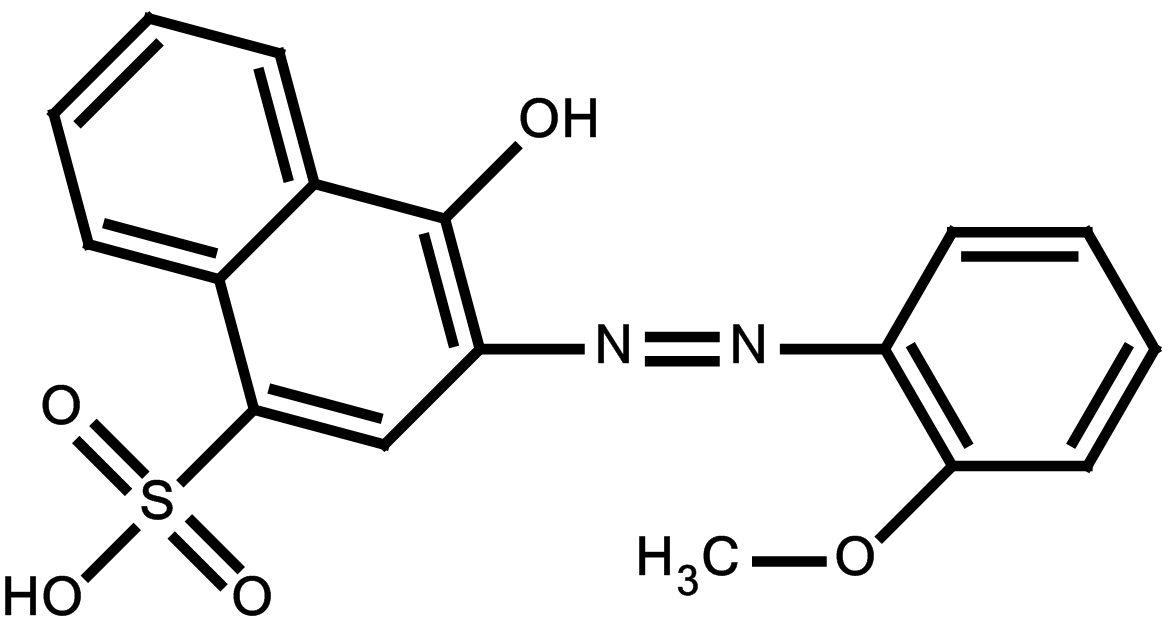  **1-naphthalenesulfonic acid,40hydroxy-3-[(2-methoxyphenyl)azo** | 0.89 | 48.92 |
| Analogs of I-85 |  |  |  |  |  |  |
| IS-70 | 156843 | 526 | 46.44±1.486 | 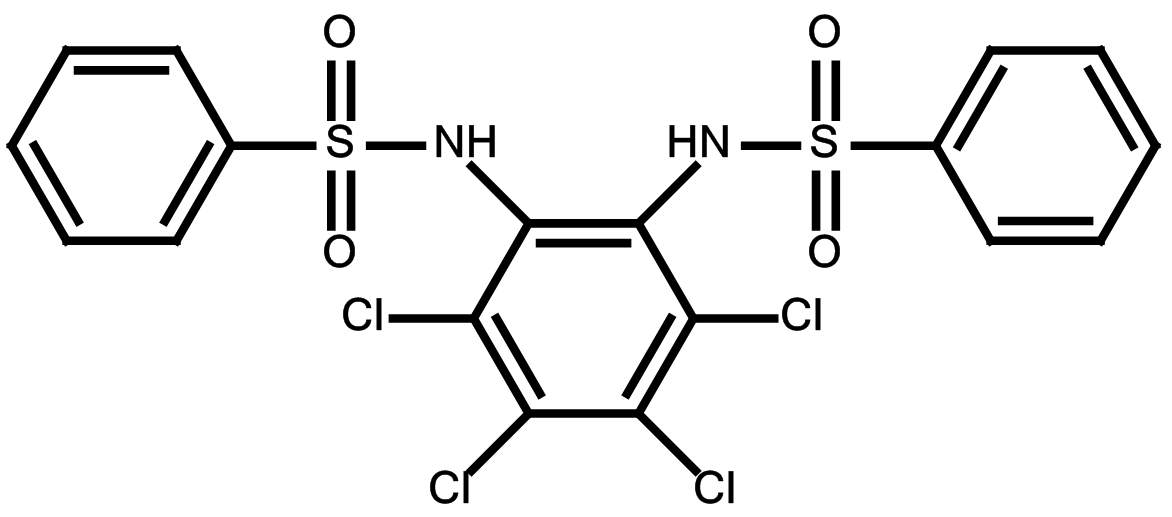  **N,N-(3,4,5,6-tetrachlorobenzene-1,2-diyl) dibenzenesulfonamide** | 0.88 | 88.28 |
| Analogs of I-103 |  |  |  |  |  |  |
| IS-47 | 5478 | 316 | 84±1.2 | **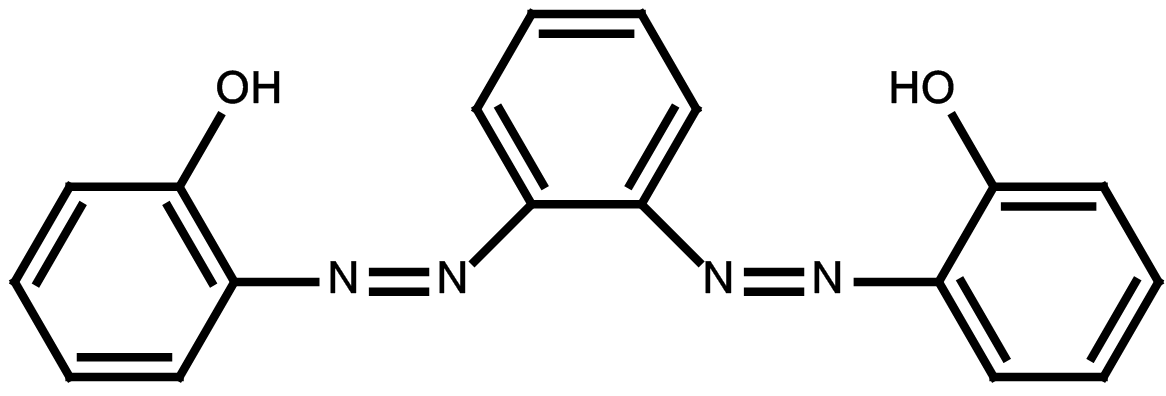**  **Phenol 2,2-[1,2-phenylenebis(nitrilomethylidyne)bis** | 0.82 | 265.82 |
| IS-51 | 22596 | 229 | 16.52±  1.72 | 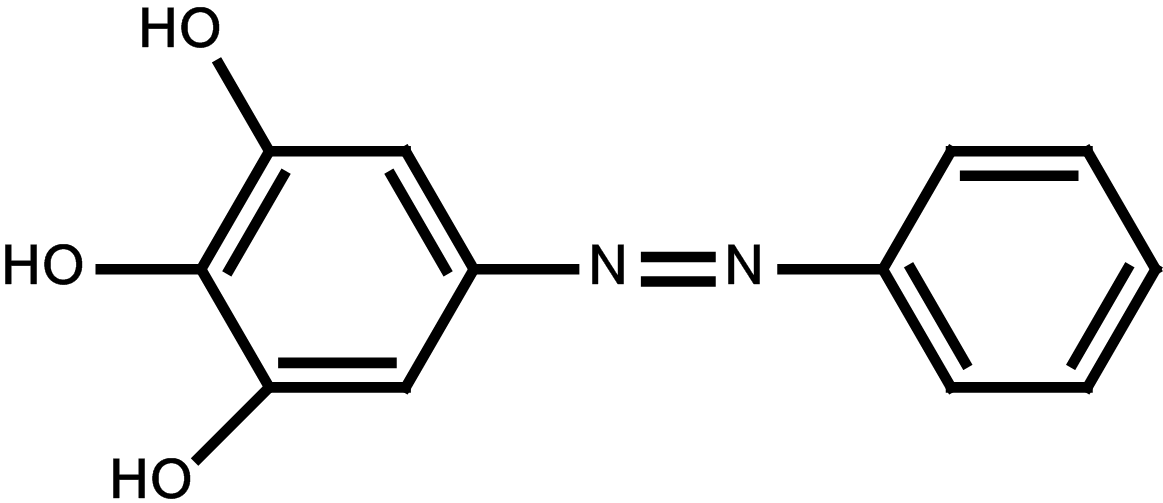  **5[phenylimino)methyl]benzene-1,2,3 triol** | 0.81 | 72.05 |
